# Light‐Harvesting Nanomaterials Based on Dyes for Energy Transfer and Amplified Biosensing

**DOI:** 10.1002/adma.202501237

**Published:** 2025-08-05

**Authors:** Andrey S. Klymchenko, Deep Sekhar Biswas, Pascal Didier

**Affiliations:** ^1^ Laboratoire de Bioimagerie et Pathologies UMR 7021 CNRS ITI SysChem Faculté de, Pharmacie Université de Strasbourg Illkirch 67401 France

**Keywords:** amplified sensing, energy transfer, light‐harvesting materials, multi‐chromophore systems, nanoparticles

## Abstract

Fascinating process of light harvesting (LH) in plants and bacteria leads to photosynthesis of oxygen and organic matter on the planet. It inspires researchers in the last decades to develop synthetic analogues – artificial LH nanomaterials. Here, LH nanomaterials based on dyes (organic chromophores) are reviewed. The fundamental aspects of LH include dye assembly into materials with minimal energy losses (i.e., high fluorescence quantum yield with minimized aggregation‐caused quenching) and fast excitation energy transfer with large exciton migration length. The efficient energy transfer from LH nanomaterial to an acceptor leads to an amplification of acceptor emission – antenna effect, which constitutes the key performance parameter of LH nanoantenna. Individual classes of dye‐based LH nanomaterials are analyzed: covalent molecular arrays of dyes (e.g., dendrimers and macrocycles), aggregates of “classical” dyes, aggregation‐induced emission nanomaterials, metal‐organic frameworks, ion‐associated nanomaterials, and hybrid dye‐based organic systems, such as dye‐biomolecule hybrids, micelles and supramolecular polymers, dye‐loaded silica and polymeric nanoparticles. The design principles and key performance characteristics of their selected (non‐exhaustive) examples are analyzed. Due to their capacity to amplify the acceptor fluorescence signal, LH nanomaterials are particularly suitable for amplified sensing of small molecules, ions, biomolecules, which is particularly attractive for biomedical applications.

## Graphical abstract

Light harvesting (LH) in plants inspires researchers to develop artificial LH nanomaterials. Here, LH nanomaterials based on organic dyes are reviewed, considering fundamental challenges on aggregation‐caused quenching, excitation energy transfer, and exciton migration length. The efficient energy transfer from LH nanomaterial to an acceptor leads to signal amplification (antenna effect) important for sensing.

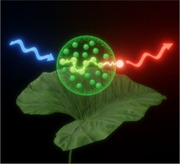

## 1 Introduction

**Figure 1:**
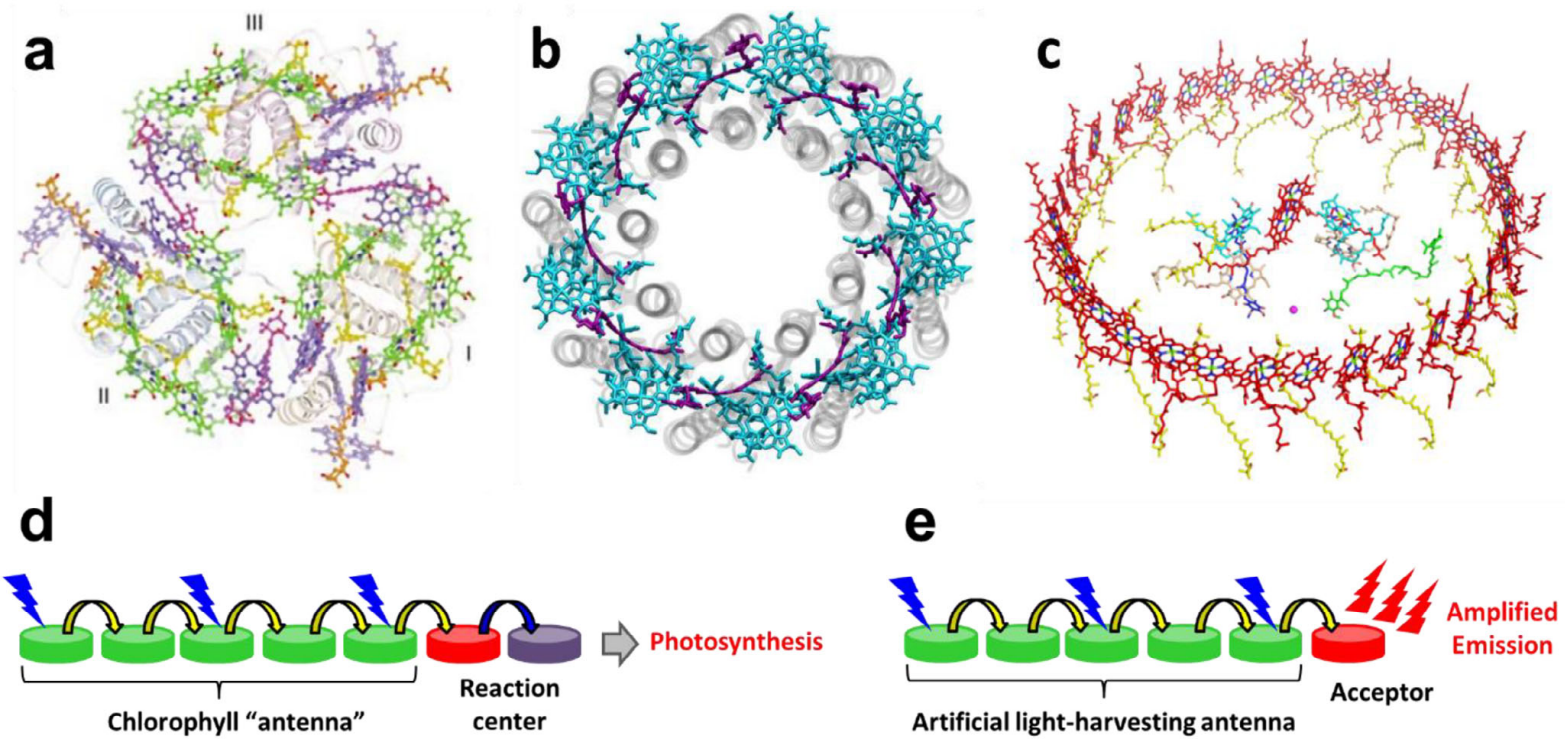
a) Structure of LHC‐II trimer complex in high plants (spinach). Monomers are labelled I–III. For clarity, the chlorophyll phytyl chains and lipids are omitted. Green, Chlorophyll a; blue, Chlorophyll b; yellow, lutein; orange, neoxanthin; magenta, xanthophyll‐cycle carotenoids. Reproduced with permission.^[^ ^24^ ^]^ Copyright 2004, Springer Nature. b) Structure of the purple‐bacterial peripheral antenna complex (LH2) from Rhodoblastus acidophilus. Top view with regard to the bacterial membrane plane, seen from the cytoplasm. The protein backbone is shown in grey. Bacteriochlorophyll a is shown in cyan, rhodopin glucosides (carotenoids) in purple. Reproduced with permission.^[^ ^25^ ^]^ Copyright 2006, Elsevier. c) Structure overview of the LH1‐RC complex from Rhodospirillum rubrum. Tilted view of the cofactor arrangement. Reproduced with permission.^[^ ^26^ ^]^ Copyright 2020, Springer Nature. Schematic illustration of light harvesting process in natural antenna (d) and in artificial LHS leading to amplified emission of the acceptor (e).

Light harvesting (LH) and electronic energy transfer are ubiquitous phenomena in nature that occur when light interacts with matter. They are an essential part of photosynthesis, a process responsible for life on our planet, converting solar light energy into the energy of chemical bonds. Plants use the energy transfer in so‐called light‐harvesting complexes (including chlorophyll) of leaves to efficiently collect light energy and deliver it to the photosynthetic reaction center. Both in plants and cyanobacteria, these complexes present multiple repeats of chromophores perfectly organized in space by a protein scaffold (**Figure** **1**a–c). This well‐defined assembly of chromophores ensures that the energy is efficiently harvested and then transferred to the photosynthetic center with minimal energy losses (Figure 1d). The efficiency of harvesting is directly linked to the capacity of the individual chromophores to absorb light (molar absorption coefficient) and the total number of those chromophores in the antenna. On the other hand, the energy transport is ensured by so called excitation energy transfer (EET) or exciton migration, mainly following so‐called Förster and Dexter mechanisms, explained in the next chapters. By tuning the number of those LH antennas, the plants and bacteria adapts to the environment and ensure that photosynthesis can take place even under very low‐light conditions. These natural devices inspired scientists to develop nanomaterials that operate as artificial light‐harvesting systems (LHS).^[^ ^1^, ^2^, ^3^, ^4^ ^]^ Development of artificial LH systems has been a long journey. An important example is conjugated polymers, where the aromatic units are connected by conjugating π‐bonds into polymers. In these structures excitation energy is delocalized within the conjugated chain, thus ensuring efficient energy transport. These systems, in the form of individual polymeric chains or nanomaterials, represent probably the vastest family of artificial LHS. As they have already been extensively reviewed,^[^ ^5^, ^6^, ^7^, ^8^, ^9^ ^]^ they will be out of the scope of the present review. Here, we would like to focus on the LH systems built from small‐molecule organic chromophores (or dyes). Probably the most classical examples of LH systems based on dyes are dye arrays in form oligomers, macrocycles, and dendrimers, where the dyes are brought together by covalent strategies.^[^ ^10^, ^11^, ^12^, ^13^, ^14^ ^]^ The close proximity of dyes connected by the covalent bonds, ensured ultrafast EET on the picosecond time scale and thus energy transport through multiple chromophores. However, as these systems are limited by the number of dyes that could be connected within the macromolecule, supramolecular approaches emerged in the last decades, which resulted in the vast variety of remarkable classes of self‐assembled LH nanomaterials. These include nanomaterials based on classical organic dyes, aggregation‐induced emission materials, metal‐organic frameworks, and ion‐associated materials. Finally, dye‐based hybrid materials are built with the help of specific molecules/matrixes (e.g., surfactants, biomolecules, polymers or inorganic porous materials). These materials include micelles and supramolecular polymers, complexes/conjugates of dyes with nucleic acids and proteins, dye‐loaded silica and polymeric NPs, etc. All these systems will be described in detail focusing on their design and LH properties. It should be noted that one common feature of all these systems is their capacity to fluoresce. Indeed, as these systems feature minimal energy losses, their excitation energy is dissipated to a large extent due to fluorescence emission, which allows their characterization by powerful fluorescence techniques as well as multiple applications. As the major focus of the review is dye‐based systems, we will also not present here LH nanomaterials based on inorganic systems, which were well reviewed for semiconductor quantum dots,^[^ ^15^ ^]^ perovskites,^[^ ^16^, ^17^ ^]^ upconversion nanomaterials,^[^ ^18^, ^19^ ^]^ periodic mesoporous organosilica and organoclay,^[^ ^20^ ^]^ etc.

The applications of artificial LH systems are vast, ranging from photovoltaics and artificial photosynthesis to chemical and biological sensing. Optical sensing, especially fluorescence biosensing, is a rapidly expanding area of science and technology with potentially enormous impact on society, because it enables the detection of (bio)molecules in the environment, food, and clinical samples. Biosensing becames particularly important in the rapid detection of disease markers for diagnostics, due to raising challenges related to cancer and viral pandemics (notably COVID‐19). To this end, specially designed biosensors or probes, based on emissive molecules (dyes), nanomaterials, and bulk materials have been developed, and many of them exploit electronic energy transfer, e.g. Dexter, radiative and coherent transfer as well as Förster resonance energy transfer (FRET). Among these, FRET between the donor and acceptor is probably the most common mechanism to ensure the response of molecular probes to the analyte.^[^ ^21^ ^]^ As the FRET process depends on the donor‐acceptor distance at the nanometer scale, it is a universal “ruler” that enables monitoring almost any sort of molecular and biomolecular interactions.^[^ ^22^ ^]^ Therefore, FRET is a powerful transduction mechanism in biosensors and nanosensors, where a molecular recognition process is coupled to a change in donor‐acceptor distance, and thus triggering a fluorescence response.^[^ ^15^, ^22^ ^]^

The key problem of existing optical (fluorescence) biosensors is their insufficient sensitivity, caused by the limited brightness of molecular emitters (defined as a product of the molar absorption coefficient and the quantum yield).^[^ ^23^ ^]^ Without specially designed schemes of molecular amplification (like PCR based on enzymes), fluorescent molecular probes cannot surpass a limit of detection in the nanomolar regime, whereas bio‐detection in clinical diagnostics applications usually requires orders of magnitude higher sensitivity. In a FRET‐based molecular probe, a single molecular recognition event triggers the response of only one fluorescent donor/acceptor dye, which is by far not enough to detect sub‐nanomolar concentrations of the target. Moreover, in complex biological media, the weak signal from a molecular probe is hindered by a strong background.

New opportunities are offered by LH fluorescent organic materials, especially fluorescent nanoparticles, which can be >100‐fold brighter than single dyes^[^ ^23^ ^]^ and therefore, can drastically increase the sensitivity of existing biosensors. In the ideal nanoparticle‐based biosensor, a single molecular recognition event should switch FRET on or off for the whole particle. This would result in so‐called signal amplification, where one event triggers a response equivalent to hundreds of fluorescent dyes. Light harvesting and efficient FRET can also result in amplification of the acceptor emission, so‐called antenna effect, where photons collected by multiple donors are shuttled to the single acceptor (Figure 1e). If this acceptor is a fluorescent probe, antenna effect can largely improve its sensitivity by increasing signal to noise ratio. However, achieving strong optical amplification in FRET assays using nanomaterials remains a challenge because nanoparticles are much larger than single molecules and thus, less effective as FRET partners. Inspiration to solve this problem comes from the light‐harvesting principle realized by natural LH systems that bring together hundreds of porphyrin dyes in complexes with proteins. It ensures efficient dye‐dye communication by fast EET, allowing energy transport through long distances providing the mechanism for efficient FRET from multiple donors to a single acceptor within nanomaterials (Figure 1e). In the present review, we analyze and combine the concepts LH nanomaterials and the electronic energy transfer based on a rapidly growing number of examples of emissive dye‐based materials and explore their potential as advanced nanoprobes for amplified biosensing.

## 2 Basics of Multi‐Chromophore Organic Systems

### 2.1 Classification of Multi‐Chromophore Materials

The multi‐chromophore systems are ubiquitous spanning from natural to synthetic materials. In nature, they represent light‐harvesting complexes in bacteria and plants, which are composed of porphyrins confined at the nanoscale with specific proteins (Figure 1a–c). Their precise organization in space ensureslow energy losses after light excitation as well as efficient energy transport toward the photosynthetic center (Figure 1d). On the other hand, a multitude of synthetic optical organic materials are built from organic dyes. Primarily, the dyes can be organized in form of crystals either alone or in complex with metal ions, e.g. in metal organic frameworks. They can be also in form of amorphous solids, for instance in case of ion‐associated materials. All these dye‐based structures can take the form of bulk materials or nanoparticles, which depend on the preparation methods. Another important family of multi‐chromophore organic systems are blended (hybrid) materials, where the dyes are assembled within a matrix (organic or inorganic), for example in case of micelles, dye‐loaded silica or polymeric nanoparticles, etc. In contrast to pure dye (nano)materials, the blended materials ensure better control of formed nanostructures, their stability and surface chemistry, which are particularly important in biological applications. However, in the assembled (aggregated) form, dyes behave very differently from those in the solution (molecular form). Thus, interaction within the fluorophores in the ground and/or in the excited state, which leads to new properties. In the ground state, the formation of J‐aggregates and H‐aggregates (see chapter 2.3) results in a change in the absorption and emission spectra. In the excited state, dyes can form homodimers (excimers) or heterodimers (exciplexes),^[^ ^2^, ^3^, ^4^ ^]^ all characterized by different emission spectra compared to those in solution. Generally, dye‐dye interactions lead to the loss of fluorescence (quenching), although there are remarkable exceptions to this general rule. In particular, it concerns aggregation‐induced emission (AIE) materials where aggregation leads to fluorescence enhancement.^[^ ^5^ ^]^ Finally, confining the dyes in the nanoscale lead to energy transfer processes, where the energy can migrate through multiple dyes within the (nano)materials spanning to relatively long distances. This phenomenon is particularly important in light harvesting where a large ensemble of dyes can transfer energy toward functional acceptor (Figure 1d,e). This phenomenon ensures efficient energy collection and further conversion, being crucial for application in artificial photosynthesis as well as in biosensing.

### 2.2 Self‐Quenching in Multi‐Chromophore Materials

Fluorescent chromophores (fluorophores) at higher concentrations generally lose their emission because of their aggregation and this phenomenon is known as self‐quenching or aggregation‐caused quenching (ACQ).^[^ ^27^, ^28^, ^29^ ^]^ ACQ is one of the main challenges associated with the multi‐chromophore systems. This problem occurs from the strong excitonic coupling when dyes are packed together, resulting in forbidden transitions (e.g., in case of H‐aggregates, see below).^[^ ^30^, ^31^ ^]^ Other reasons of ACQ in multi‐chromogenic systems include excimer formation, excited‐state intermolecular charge and electron transfer within the aggregated dyes as well as reabsorption within large dye ensembles.^[^ ^32^ ^]^ Quenching in multi‐chromophore materials can also happen at the level of individual dyes, through classical processes, such as intersystem crossing leading to formation of non‐emissive triplet state, photo‐generated radical states, twisted excited state charge transfer and intramolecular electron transfer.^[^ ^32^ ^]^ Both intermolecular and intramolecular quenching processes are responsible for the formation of non‐emissive states of the dyes in materials, which are also called “dark states”. The larger fraction of the dark states in a given multi‐chromophore material, the lower its fluorescence quantum yield. Moreover, EET (or exciton diffusion) within the dyes^[^ ^33^, ^34^ ^]^ can further enhance quenching phenomenon. First, a large ensemble of emissive dyes coupled by EET can be efficiency quenched by a single dark state, which could function as an energy trap (sink) for the diffusing excitons.^[^ ^35^, ^36^ ^]^ Second, two migrating excitons can quench each other on collision, for instance, by singlet‐singlet^[^ ^37^ ^]^ or singlet‐triplet annihilation.^[^ ^37^, ^38^ ^]^ Decades of research are going on to address the problem of ACQ in order to obtain bright fluorescent (nano)materials,^[^ ^23^ ^]^ which will be explained below in chapters 2.4 and 4.

### 2.3 H‐ and J‐Aggregates

**Figure 2:**
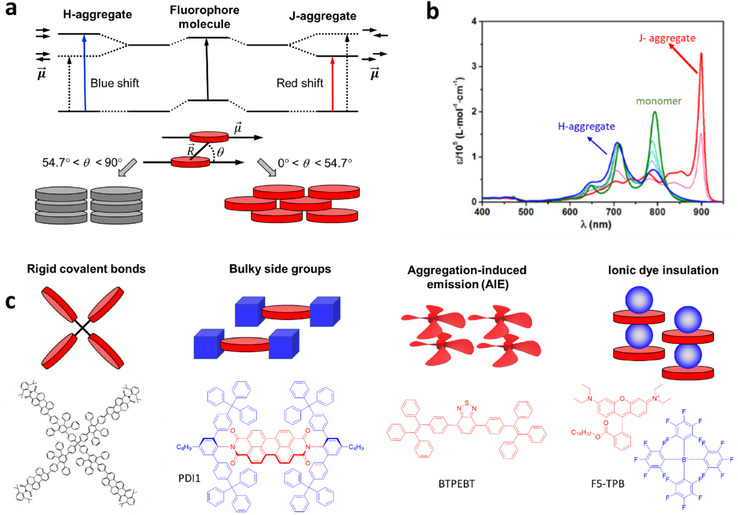
a) The excitonic orbital splitting of dye dimers at different angles. The solid arrow shows allowed transitions and the dotted arrow shows forbidden transitions. Reproduced with permission.^[^ ^23^ ^]^ Copyright 2023, the Royal Society. b) The absorption spectra of aggregates and monomers. Reproduced with permission.^[^ ^44^ ^]^ Copyright 2018, American Chemical Society. c) The most widely used strategies to prevent ACQ for fluorescent dye‐based materials. Reproduced with permission.^[^ ^23^ ^]^ Copyright 2023, the Royal Society.

The self‐association of dyes in solution or at the solid‐liquid interface is often seen with increasing concentration of dyes because of very strong intermolecular forces, such as van der Waals, electrostatic, hydrophobic and π‐π interactions.^[^ ^39^, ^40^ ^]^ Two extreme cases of dye‐dye aggregates should be highlighted. First, head‐to‐tail assemblies of dyes correspond to J‐aggregates, which are characterized by a sharp and red‐shifted absorption band. Second, the face‐to‐face assemblies of dyes correspond to H‐aggregates, characterized by a blue shifted absorption spectrum (**Figure** **2**a,b).^[^ ^41^, ^42^, ^43^ ^]^

According to the exciton theory, adapted by Kasha to organic fluorophores,^[^ ^43^, ^45^ ^]^ dye molecules are regarded as point dipoles, and the excitonic states of the aggregates split into two levels by the interaction between the transition dipoles. Due to the symmetry selection rules, for H‐aggregates only S_0_ to S_2_ transition is allowed, whereas for J‐aggregates S_0_ to S_1_ transition is allowed (Figure 2a). The S_0_ to S_2_ transition of H‐aggregate is higher in energy than monomeric transition, whereas for J‐aggregate, S_0_ to S_1_ transition is lower in energy than that of monomer which is the origin of the blue and red shift in the absorption spectra, respectively (Figure 2b).^[^ ^44^ ^]^ To describe the H‐ and J‐aggregates, Kasha used coulombic intermolecular coupling within the framework of Frankel exciton theory. When the two point‐dipoles (1 and 2) interact, then coulombic dipole‐dipole coupling ($J_{Coul}^{pd}$) can be expressed as:

(1) $$J_{Coul}^{pd}=\frac{\mu_{1}.\mu_{2}-3\left(\mu_{1}.\hat{r}\right)\left(\mu_{2}.\hat{r}\right)}{4\pi \varepsilon r^{3}}\;$$

where μ_1_,μ_2_ are the dipole moments of the molecules 1 and 2 (S_0_‐S_1_ transition), $\vec{r}=r.\;\hat{r}$ is the displacement vector between the dipoles and ε is the dielectric constant of the medium. For dimers, this equation reduces to

(2) $$J_{Coul}^{pd}=\frac{\mu^{2}\left(1-3cos^{2}\theta \right)}{4\pi \varepsilon r^{3}}$$

The optical properties of the aggregates depend on the relative distance and the angle between dipoles (θ). J‐aggregates are characterized by a conformation for which θ < 54.7°, while H‐aggregates conformation corresponds to θ >54.7°. These configurations result in two delocalized excited states with an energy difference of 2$J_{Coul}^{pd}$. For J‐aggregates, the symmetric state has lower energy (red shift, optically allowed transition), whereas for H‐aggregates, the antisymmetric state has lower energy (blue shift, optically forbidden transition, Figure 2a,b).^[^ ^43^, ^44^, ^45^ ^]^

In addition to the absorption, Kasha also showed that radiative decay rate is different for these two types of aggregates. In H‐aggregates, there is a rapid vibrational relaxation in excited state allowing to reach the ground state. As this state has out‐of‐plane alignment of the transition dipole, its transition to ground state is symmetry forbidden. For J‐aggregates, the transition from the lowest energy excited state (in plane) to ground state is allowed due to symmetry. The transition dipole in the J‐aggregate increases by the factor of $\sqrt{2}$ compared to monomer.^[^ ^43^, ^45^ ^]^ The fluorescence of the J‐ and H‐aggregates also depends on the relative distance and the angle associated with the dipoles. Therefore, intermediate aggregation states, such as distorted H‐aggregates, can be highly emissive.^[^ ^46^, ^47^, ^48^ ^]^

### 2.4 Methods to Enhance Emission in the Multi‐Chromophore Systems

The quenching processes, in particular due to formation of H‐aggregates, decrease fluorescence brightness of materials and block the energy transfer processes because the dark states function as energy traps. Therefore, fighting ACQ is essential for preparation of bright fluorescent materials characterized by efficient energy transfer and optimal light‐harvesting properties. Formation of J‐aggregates is the most traditional way,^[^ ^41^ ^]^ but it is rarely used because it is difficult to control in nanomaterials.^[^ ^49^ ^]^ Therefore, several alternative approaches to minimize ACQ in multi‐chromophore systems were developed. Encapsulation of dyes into nanomaterials leads to separation of dyes in the matrix of polymer, lipid or porous inorganic material, such as silica or zeolite. Moreover, the smaller number of degrees of freedom restricts the dyes to relax through non‐radiative pathways, which eventually helps them to have better QY.^[^ ^29^ ^]^ However, generally attempt to increase dye loading in these blended systems leads to the strong quenching already at 1 wt% loading.^[^ ^29^, ^50^ ^]^ Therefore, dedicated design of dyes should be done to ensure low ACQ at high dye concentration in nanomaterials (Figure 2c). The purely synthetic organic chemistry approach is to combine the dyes within a dendrimer, where fluorophores cannot stack because of rigid covalent bonds (Figure 2c).^[^ ^11^, ^14^, ^51^, ^52^, ^53^ ^]^ This method is efficient but challenging in terms of synthesis and it is limited in terms of number of dyes per dendrimer. The other classical approach is introduction of bulky side groups that prevent dye π‐stacking and thus reduce the probability of ACQ.^[^ ^54^, ^55^, ^56^ ^]^ This approach generally requires multistep synthesis leading to highly complex molecular architectures with out of plane substituents, as exemplified by PDI derivatives (Figure 2c).^[^ ^57^, ^58^ ^]^ A vast progress in the field of highly emissive nanomaterials has been made since the aggregation‐induced emission (AIE) concept was introduced by Tang and co‐workers.^[^ ^28^, ^59^, ^60^ ^]^ The designed AIE dyes (AIEgens) are structurally flexible propeller shaped dyes that undergo efficient non‐radiative deactivation in the excited state due to rotational or vibrational relaxation (Figure 2c).^[^ ^61^ ^]^ The aggregated state leads to restriction of intermolecular rotation/vibration of these dyes as well as to a particular dye‐dye assembly, intermediate between H‐ and J‐aggregates, yielding bright fluorescence in the solid state. An alternative vision on ACQ was to shift the focus on the dye counterion in ionic dyes (Figure 2c). Bulky ions can act as a spacer between dye molecules to weaken the intermolecular dye‐dye interaction and enhance the quantum yield (QY).^[^ ^23^ ^]^ It was originally shown by Yao and Warner that bulky ions can effectively reduce the effect ACQ in pure dye salts.^[^ ^47^, ^62^ ^]^ Our works further extended this concept to blended polymeric materials, dye‐loaded polymeric nanoparticles,^[^ ^36^, ^63^, ^64^ ^]^ which are considered among the brightest nanomaterials, reported to date.^[^ ^23^ ^]^ Recently, Zhang and co‐workers proposed to reduce ACQ by co‐assembly of polycyclic aromatic hydrocarbons (dyes) with octafluoronaphthalene.^[^ ^65^ ^]^ The latter also serve as a molecular barrier between the dyes, thus minimizing ACQ, but in this case for neutral dyes in co‐crystals. The specific examples of multi‐chromogenic materials with reduced ACQ will be described in later chapters dedicated to LH materials.

## 3 Energy Transfer, Light‐Harvesting, and Signal Amplification

Energy transfer takes place when two chromophores are located at sufficiently short distances on the scale of nanometers. The excitation energy is transferred non‐radiatively from the dye in the excited state to the dye in the ground state. In multi‐chromophore materials, one should consider energy transfer within the same dyes (homotransfer) or different dyes (heterotransfer). Two types of energy transfer are important in multi‐chromophore systems: Dexter and Forster.

### 3.1 Dexter Energy Transfer

Dexter type of energy transfer involves the direct exchange of electrons between closely situated donor and acceptor entities.^[^ ^66^ ^]^ This is a short‐range, radiationless energy transfer mechanism that involves a direct exchange of electrons between an excited donor and a ground‐state acceptor. Unlike FRET, which is mediated by long‐range dipole–dipole coupling, Dexter transfer relies on overlap of the electronic wavefunctions and becomes dominant when spin‐forbidden transitions are involved, such as triplet–triplet energy transfer.^[^ ^67^, ^68^ ^]^

First introduced by Dexter in 1953, this mechanism was proposed to explain spin‐forbidden energy transfer processes, where FRET is ineffective due to the requirement of spin conservation. In Dexter transfer, energy is exchanged through electron–electron Coulomb interactions, particularly via a correlated two‐electron exchange mechanism. Additionally, higher‐order one‐electron contributions may also play a role, particularly when donor and acceptor are connected by a molecular bridge. Dexter transfer requires close proximity between donor and acceptor molecules, typically in the range of 0.5 to 1.0 nm. This proximity is necessary to ensure effective overlap of their electronic wavefunctions. As a result, the rate of Dexter energy transfer decreases exponentially with distance, following the relationship:

(3) $$k_{T}^{\mathrm{Dext}\mathrm{er}}=\frac{2\pi }{h}\mathrm{KJexp}\frac{-2r}{L}$$

where $k_{T}^{Dexter}$ is the rate constant, L is average Bohr radius, K is a constant including orbital overlap, J is the spectral overlap integral, described in a later section.

Dexter transfer is especially important in cases where spin conservation is necessary, such as in triplet–triplet energy transfer. This spin‐allowed process proceeds without requiring intersystem crossing, although FRET, which would need a spin flip for each triplet transfer step. While Coulombic (long‐range) interactions dominate in spin‐allowed singlet transitions and are the basis of Förster theory, they are strongly screened in polar environments and ineffective in spin‐forbidden cases. In such cases, Dexter's short‐range electron exchange mechanism becomes the dominant pathway for energy transfer.^[^ ^69^ ^]^ In this review, we will mainly focus on FRET, which is predominant in case of dye‐based systems.

### 3.2 Förster Resonance Energy Transfer

Energy transfer (ET) or exciton energy transfer (EET) refer to energy transfer from donors to acceptors. Among all other ET mechanisms, Förster Resonance Energy Transfer (FRET) corresponds to a weakly coupled (incoherent) resonance energy transfer process (RET) between fluorescent donors (D) and acceptors (A) when they remain in very close proximity and optimized orientation. The theory developed by Theodor Förster has been largely described in the textbooks and reviews^[^ ^22^, ^32^ ^]^ and is beyond the scope of this review. Hence, we just remind the main aspect of the FRET mechanism. The ET process results from a pure Coulombic interaction, it is a nonradiative phenomenon and depends on the relative D‐A distance (E_FRET~_ r^−6^) with a working range that is typically about 1–10 nm for conventional organic dyes (characterized by the so called Förster radius). In addition, FRET efficiency is also modulated by the spectral overlap between the donor emission and acceptor excitation together with the relative orientation of the dipoles associated to the donor and acceptor.

Experimentally, FRET efficiency can be calculated using the emission properties of the donor:

(4) $$E_{FRET}=1-\;\frac{I_{D}^{A}}{I_{D}\;}=1-\frac{\tau_{D}^{A}}{\tau_{D}}$$

where *I_D_* and $I_{D}^{A}$ are the donor emission intensities in the absence and presence of acceptor and $\tau_{D}^{A}$ and τ_*D*_ are the average lifetime in the presence and absence of acceptor.

In case when the quantification of FRET efficiency is problematic due to a lack of appropriate experimental parameters, semi‐quantitate measure of FRET efficiency, so‐called FRET ratio could be used, if the acceptor is emissive. The FRET ratio can be determined:

(5) $$FRET\;ratio=\frac{I_{A}}{I_{D}+I_{A}}$$

where *I_A_* and *I_D_* are the total emission intensity of acceptor and donor at donor excitation. FRET ratio depends on the quantum yields of the dyes as well as on FRET efficiency and therefore is not a direct measure of the FRET efficiency. It is particularly common when FRET studies are done in complex media and/or using ratiometric fluorescence imaging.^[^ ^70^, ^71^ ^]^

### 3.3 Energy Transfer in Multi‐Chromophore Systems

Systems with multiple chromophores present unique characteristics compared to single chromophore systems.^[^ ^72^, ^73^ ^]^ In addition to their photoluminescence brightness, they can exhibit collective behavior, where the dyes can communicate to each other by energy transfer and thus exhibit collective behavior.^[^ ^35^, ^36^, ^74^, ^75^ ^]^ In this respect, multi‐chromophore systems could be divided into those characterized by low and high cooperativity of dyes. In the former, the encapsulated dyes behave like independent fluorophores. In the latter case, the dyes are coupled by fast energy transfer (EET, exciton migration) through Dexter or Föster mechanisms.

#### 3.3.1 Low Cooperativity of Dyes

The typical low‐cooperativity systems are dye‐loaded nanomaterials at low dye loading. FRET theory was originally developed to describe the energy transfer between point dipoles (single donor and acceptor) but the FRET formalism can be transposed to systems containing multiple donors and acceptors. In the case of multiple (n) acceptors interacting with a single donor, the FRET efficiency can be calculated by summing over the n uncoupled FRET pathways^[^ ^76^ ^]^ and is given by the following equation:

(6) $$E_{FRET}\left(n\right)=\frac{nk_{FRET}}{nk_{FRET}+k_{other}}=\frac{\frac{nk_{FRET}}{k_{FRET}+k_{other}}}{\frac{k_{FRET}+k_{other}+\left(n-1\right)k_{FRET}}{k_{FRET}+k_{other}}}=\frac{nE_{0}}{1+\left(n-1\right)E_{0}}$$

where *E*_0_ is the FRET efficiency of single D–A pair. Upon an increase in the number of acceptors, the FRET efficiency tends toward unity but the value of the Förster radius remains the same for all pairs (considering the acceptors are at the same distance with respect to the donor).

For multiple donors and acceptor systems, Monte Carlo approaches have been used to determine the FRET efficiency between donors and acceptor randomly distributed over space in the absence of cooperativity. For instance, Danuser et al. and Rigby et al. determined the probability of acceptor FRET sensitization by m donors:^[^ ^77^, ^78^ ^]^

(7) $$P_{A}=1-{\left(1-E_{FRET}\right)}^{m}$$

However, this equation is only valid for low excitation regime where all donors and acceptors have relaxed to their ground state before the next photon excitation. Nevertheless, the ensemble is still described within the Förster formalism where several donor‐acceptor pairs are considered. This description does not account for any cooperativity between donor dyes or acceptors dyes. The particular case of energy transfer in the presence of dye cooperativity (e.g., multiple interacting donors to a single acceptor, antenna system) will be discussed with more detail in the subsequent parts.

#### 3.3.2 High Cooperativity of Dyes

In the multi‐chromogenic system with high cooperativity, the energy donor dyes are not independent. They are generally coupled by fast excitation energy transfer (EET) through Förster or Dexter mechanisms, which ensure that after initial excitation of one donor dye, the energy migrates through multiple donor dyes until it reaches the acceptor (Figure 1e) or converts into light or heat. In these systems the chromophores are located at close distances (1–5 nm), which ensures these energy migration processes. The most widely known multi‐chromogenic system with high cooperativity are the photosynthetic light harvesting complexes (LHC) in bacteria and plants (Figure 1a–c).^[^ ^75^, ^79^, ^80^ ^]^ In these systems, chromophores are arranged in an ordered geometry by proteins using non‐covalent interactions. High resolution X‐ray crystal analysis showed that the reaction center (RC) is surrounded by LHCs.^[^ ^81^ ^]^ The basic role of LHC is that of an antenna which absorbs photons and this energy, collected from large area, is transferred to the RC with high efficiency (Figure 1d). In the following part, we will discuss more about the theories of light‐harvesting systems (LHS) and antenna effect for the signal amplification.

#### 3.3.3 Natural Light‐Harvesting Systems

Over evolution on Earth, several organisms have adopted a strategy based on using light energy to produce the organic molecules they need for their metabolism. If one considers the average power density of solar light in the visible range (about 1 kW/m^2^) and the absorption cross‐section of organic chromophores (about 10^−20^ m^2^), this corresponds to absorption of about 20 photons per second. Therefore, the optimal use of solar energy requires highly efficient light harvesting systems (LHSs). One of the most important features of natural LHSs is their densely packed chromophores, which act as antenna system to collect the light energy for the photosynthesis and help organisms to survive in low light regime.^[^ ^80^ ^]^ Therefore, the common features shared by the natural LHSs relies in the high number (from 50 to 200, corresponding to local concentration up to 0.3 M) of densely packed chromophores that collect the energy and then transport it to the reaction center.^[^ ^79^ ^]^ In these systems, the typical chromophore‐chromophore distances range from 0.5 to 3 nm^[^ ^82^ ^]^ and the spatial arrangement of the chromophores is ensured by the protein scaffold that give rise to specific relative orientation between the chromophores. In particular, parallel or near‐parallel alignments are common in LHSs to facilitate unidirectional energy flow. Such geometries can give rise to exciton delocalization across multiple chromophores, reducing the likelihood of trapping and promoting rapid energy transfer (i.e., from tens of femtoseconds to hundreds of picoseconds).^[^ ^83^ ^]^ For instance, in the LH2 to LH1‐RC pathway of the purple bacteria (Figure 1a–c), an exciton delocalizes over distances of approximately 4 to 6 chromophores.^[^ ^84^ ^]^ The studies dedicated to exciton transport mechanisms evidenced the contribution of several quantum effects such as quantum coherence,^[^ ^85^, ^86^, ^87^ ^]^ quantum entanglement^[^ ^88^, ^89^ ^]^ and dephasing.^[^ ^90^ ^]^ It is widely accepted, both theoretically and experimentally, that quantum effects are present in light harvesting complexes, but there is still debate about the nature of these effects.^[^ ^91^ ^]^ In particular, a combined coherent–incoherent motion,^[^ ^92^ ^]^ such as an incoherent hopping of excitons delocalized over few units,^[^ ^93^ ^]^ has been associated with the exciton transport mechanism in natural LHSs. Nevertheless, energy transfer efficiency in single light‐harvesting complexes is exceptionally high (>90%), ensuring that most absorbed sunlight is funneled to the reaction center (over distance up to 50 nm in LH2 in plants and bacteria with an overall efficiency ranging from 50 to 90%)^[^ ^94^, ^95^ ^]^ for photochemical conversion. Inspired by the natural systems, researchers aim to develop materials containing a high number of chromophores (>100s) in a confined environment while controlling their spatial organization in order to achieve efficient energy collection together with fast long‐range EET.

#### 3.3.4 Antenna Effect and Signal Amplification

Antenna Effect (AE) is an important parameter for describing light‐harvesting properties of LHS. Formally, antenna effect means the amplification of acceptor emission due to energy transfer from multiple donors (Figure 1e).^[^ ^96^, ^97^ ^]^ There are multiple ways to calculate the antenna effect such as:

(8) $$antenna\;effect=\frac{I_{AF\lambda \left(D\right)}}{I_{AF\lambda \left(A\right)}}$$

the ratio between acceptor fluorescence intensity upon excitation of donors *I*_*AF*λ(*D*)_ and the direct excitation of acceptor *I*_*AF*λ(*A*)_.The antenna effect (AE) in the case of large donor to acceptor ratio can also be calculated from the excitation spectra as:^[^ ^98^ ^]^

(9) $$AE=\frac{I_{D-FRET\;}^{ex}-\left(I_{D}^{ex}\;\times \;f\right)}{I_{A-FRET}^{ex}-\;I_{A}^{ex}}$$

where $I_{D-FRET\;}^{ex}$ and $I_{A-FRET}^{ex}$ represent the maximum excitation intensities of donors and acceptors in FRET system respectively. $I_{D}^{ex}$ and $I_{A}^{ex}$ are the excitation intensities at the wavelengths of donor and acceptor, respectively, for a control sample with donors only. *f* is the correction factor calculated as $f=\frac{I_{D-FRET}^{em}}{I_{D}^{em}}$, where $I_{D-FRET}^{em}$ and $I_{D}^{em}$ are the emission maxima of the donors with and without acceptor in the FRET system, respectively. Antenna effect can also be estimated by knowing the number and the molar absorption coefficient of donor and acceptor molecules.

(10) $$Antenna\;effect=\frac{n_{D}\varepsilon_{D}E_{FRET}}{n_{A}\varepsilon_{A}}$$

where *n_D_* and ε_*D*_ is number and molar absorption coefficient of donors, respectively, whereas *n_A_* ε_*A*_ is number and molar absorption coefficient of acceptor, respectively, and *E_FRET_* is the FRET efficiency of the system.^[^ ^97^ ^]^

EET lies at the heart of the antenna effect in light harvesting, serving as the most fundamental mechanism that enables efficient energy capture, distribution, and utilization in light harvesting in both natural and artificial LHSs (such as dye‐based systems, conjugated polymer systems, etc.). When light strikes a light‐harvesting unit comprising hundreds of light‐harvesting pigment molecules organised in antenna complexes, EET enables the absorbed excitation energy to be swiftly and directionally transferred from high‐energy pigments (e.g., carotenoids, chlorophyll b and dyes) toward lower‐energy acceptor molecules (i.e., the reaction center). In artificial LHS, this is realized by assembly of large number of donor dyes within molecular arrays or nanoparticles, where EET ensures efficient energy to few acceptors co‐assembled within the LHS. EET, which occurs on femto‐ to picosecond timescales, is highly efficient due to the precise spatial arrangement, spectral tuning, and dipole‐dipole interactions of the pigments/dyes, which together minimize energy loss. Without EET, each reaction center would rely solely on direct photon absorption, limiting its productivity; instead, the antenna effect created by EET increases the effective cross‐section for light absorption by orders of magnitude. This not only enhances the global efficiency but also enables light harvesting in diverse and fluctuating light environments by adjusting the size or the composition of their light‐harvesting apparatus to match energy demands.^[^ ^75^ ^]^ In addition to the antenna effect, it is important to discuss the signal amplification as well. The signal amplification is mostly discussed for plasmonic systems as metal enhanced fluorescence (MEF) in literature. This enhancement of fluorescence was studied since 1980.^[^ ^99^ ^]^ There are mainly two reasons for this enhancement: (i) metal is responsible for enhancing locally the excitation field on the fluorophore by forming a hotspot. This was nicely described later for bowtie nanoantenna, where the light gets concentrated into a small gap (subdiffractional volumes) resulting in the formation of a hotspot and thus effectively excites chromophores at that position with higher brightness and amplification.^[^ ^100^ ^]^ (ii) The hotspot also increases the radiative decay rate (γ_*m*_) of the fluorophore and therefore the fluorescence emission intensity.^[^ ^101^ ^]^

The fluorescent enhancement (*f_E_*) in plasmonic systems can be calculated as

(11) $$f_{E}=\frac{QY_{m}}{QY_{0}}\frac{{\left|\mu_{if}\cdot E_{loc}\right|}^{2}}{{\left|\mu_{if}\cdot E_{0}\right|}^{2}}$$

It is evident that the enhancement depends on the electric fields (*E*_0_ the incident electric field and *E_loc_* is the local electric filed) and on the quantum yield (QY) and this quantum efficiency can be calculated as for $QY_{m}=\frac{\gamma +\gamma_{m}}{\gamma +\gamma_{m}+k_{nr}+k_{q}}$ and for $QY_{0}=\frac{\gamma }{\gamma +k_{nr}+k_{q}}$ where γ is radiative rate constant, *k_nr_* and *k_q_* are non‐radiative and quenching rate constants.^[^ ^102^ ^]^

Equation 11 shows that the fluorescence enhancement depends on the quantum efficiencies and electric field and therefore a slight changes in these parameters can modulate the response of the system based on DNA origami.^[^ ^103^, ^104^, ^105^ ^]^ These systems enabled signal amplification up to 5000‐fold for poor fluorophores and about 300‐fold good (highly emissive) fluorophore.^[^ ^104^, ^106^ ^]^ For “good” fluorophores with a high QY (>30%), the amplification originates mainly from the enhanced absorption of photons due to the plasmonic antenna. For fluorophores with a lower QY, a faster emission rate near metal surfaces increases their fluorescence QY and thus additionally contributes to fluorescence enhancement.

In contrast to plasmonic nanoantennas, artificial LH nanoantennas operate by FRET from multiple donor dyes to a single acceptor. Their signal amplification is directly linked to the antenna effect, which is generated by the efficient collection of light energy by multiple donors and then fast transport of the excitons within the LH system toward to the acceptor (Figure 1e). However, previously, the values of amplification by artificial LH nanoantennas were significantly lower compared to those achieved by the plasmonic systems. In 2017, we reported giant light‐harvesting nanoantenna based on dye‐loaded polymeric NPs.^[^ ^98^ ^]^ It exploited high cooperativity of the encapsulated fluorophores due to fast EET (30 fs time scale), with antenna effect 1150 for good fluorophores (see below). However, further investigations are needed to understand better the mechanisms in multi‐chromophore systems in order to further improve performance of artificial LH systems and expand the scope of their applications.

## 4 Light‐Harvesting in Dye‐Based Materials for Energy Transfer

By analogy with natural LH systems, creating artificial light harvesting systems requires considering several key points.^94^ (i) The donors should be densely packed to enhance light absorption (collection) and facilitate ultrafast energy transfer. (ii) The donors in the densely packed systems should not lose energy through ACQ. (iii) There should be high donor to acceptor ratio in order to achieve high values of the antenna effect. Indeed, unlike classical FRET system with donor‐to‐acceptor ratio of 1:1, in LHS this ratio should be as high as possible, so that the large number of donors collect efficiently the light energy and transfer it to few acceptors. To this end, donor dyes are generally combined together within covalent molecular arrays or nanoparticles. As mentioned above, tight assembly of donor dyes eventually results in self‐quenching due to the so‐called ACQ. Therefore, the path to efficient LH systems primarily goes through designing nanomaterials with highly dense dye packing, minimized ACQ and maximized EET. Below we describe main examples of dye‐based organic nanomaterials, designed as LH nanoantenna.

### 4.1 Covalent Molecular Arrays of Dyes

**Figure 3:**
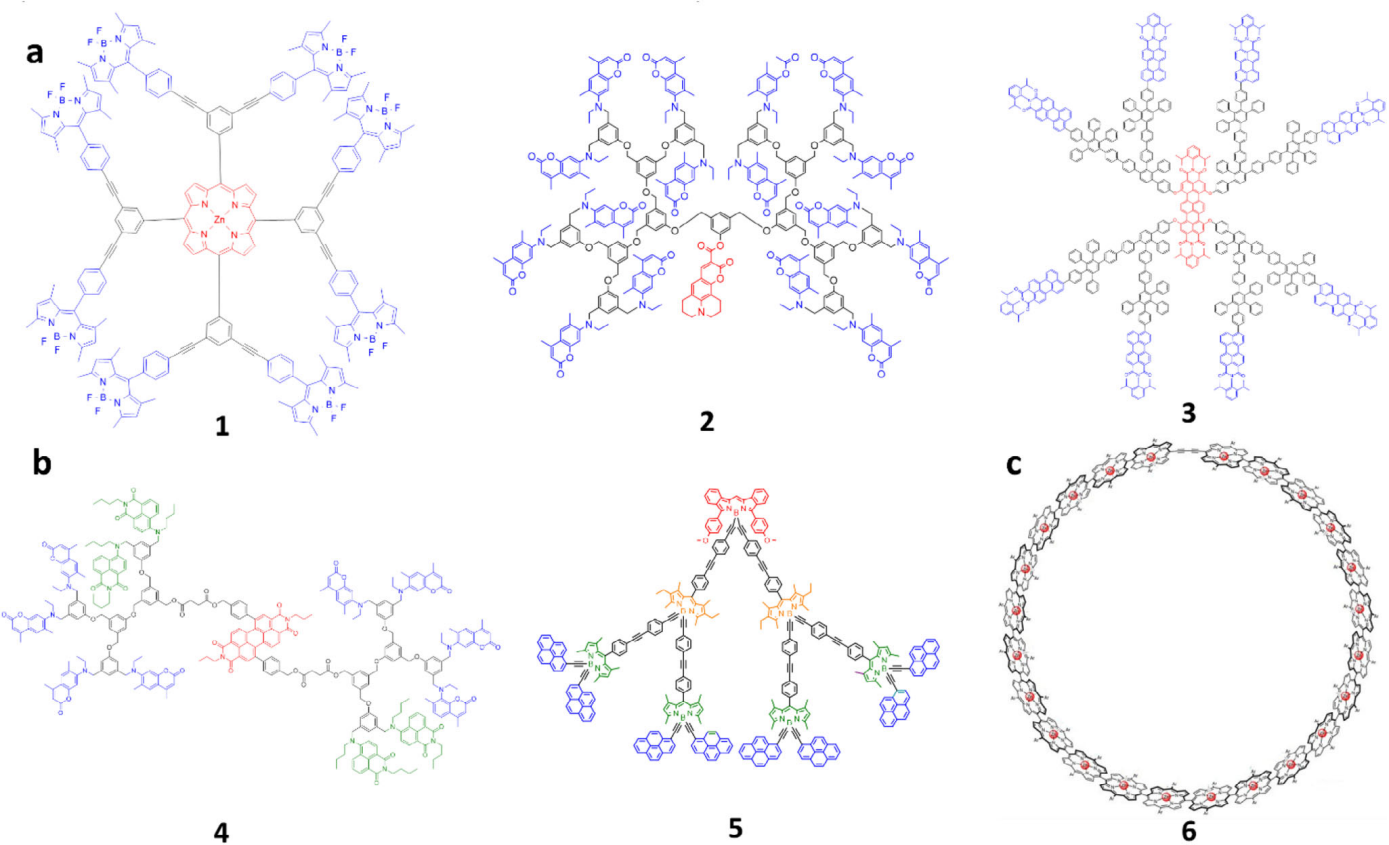
Examples of LHS based on dendrimers (a,b) and macrocycles (c). The examples include two‐component LHS (a) and multicomponent systems with cascade FRET (b). c) Chemical structure of porphyrin nanobelt. Reproduced with permission.^[^ ^118^ ^]^ Copyright 2022, Springer Nature.

Covalent arrays of dyes, dendrimers and macrocycles in particular, were the primary classical examples of artificial LHS. One of the reasons is that covalent bonds provide absolute control of chemical composition (i. e. donor‐acceptor ratio) and control of dye‐dye spacing and orientation. The latter is essential to ensure efficient energy transfer and minimized ACQ. There are excellent early reviews describing this concept,^[^ ^11^, ^14^, ^51^, ^52^, ^53^ ^]^ whereas we will focus on some selected examples. Lindsey and co‐workers introduced array of 8 BODIPY donor dyes (**1**, **Figure** **3**) built on the porphyrin scaffold as an acceptor.^[^ ^107^ ^]^ This LHS showed 80–90% FRET efficiency, which can be explained by close proximity of donors to the acceptor. Frechet and co‐workers reported an array of 16 coumarin donors (**2**, Figure 3) (G4 generation), assembled using aromatic AB2 building block together with one red‐shifted coumarin acceptor.^[^ ^108^ ^]^ The obtained LHS showed no self‐quenching of the donor dyes and nearly quantitative FRET efficiency (93%) with overall quantum yield of 40% and high extinction coefficient 184000 M^−1^ cm^−1^). Taking Equation 10, the antenna effect in this system was 4.3. Another classical example was rylene‐based dendrimers (**3**, Figure 3), containing 8 donors and a single acceptor in the core and featuring high structural rigidity and photostability.^[^ ^109^ ^]^ The obtained LHS quantum yield was 9% and overall extinction coefficient of the donors reached 258,000 M^−1^ cm^−1^. It showed nearly quantitative FRET efficiency on time scale <100 ps, which can be explained by short donor‐acceptor distance (3.1 nm).^[^ ^110^ ^]^ Here, Equation 10 suggests the value of AE of 3.0. The other level of complexity is added in cascade FRET systems, where the energy is transferred from the highest energy donor to the intermediate acceptor and finally to the final acceptor. The early example was shown by Frechet and co‐workers, where a dendrimer was built of 8 coumarin donors, four naphthilimide units as intermediate acceptors and a single perylene‐diimide acceptor (**4**, Figure 3).^[^ ^111^ ^]^ This approach ensured efficient (95%) energy transfer from high energy coumarin to perylene dye, despite relatively poor spectral overlap between these two dyes. The other remarkable example was shown by Ziessel, Harriman and co‐workers, where 4‐component energy transfer system was reported: 8 pyrene donors were connected to 4 BODIPY intermediate acceptors and then to 2 intermediate red shifted BODIPYs and finally to a single most red‐shifted BODIPY derivative (**5**, Figure 3).^[^ ^53^, ^112^, ^113^ ^]^ This configuration ensured ultrafast cascade FRET through rather long distance at the 3–150 ps time scale.^[^ ^112^ ^]^ The specific feature of those systems is exceptionally large FRET efficiency and Stokes shift, whereas antenna effect in these system is limited because of lower number of used acceptors and their limited extinction coefficient.

In order to mimic closer the natural LHS, Osuka, Kim and coworkers developed a series of multi‐porphyrin macrocycles and studied their EET,^[^ ^13^ ^]^ which included cycles containing 4–8,^[^ ^114^ ^]^ 12,^[^ ^115^ ^]^ 24^[^ ^116^ ^]^ and 32^[^ ^117^ ^]^ phorphyrin units. The cycles with particularly short chromophore‐chromophore spacing^[^ ^114^ ^]^ showed remarkably fast EET on the time scale of 300–700 fs, which was close to the time scale observed in B850 chromophore of the natural LH2 complex (100‐200 fs).^[^ ^13^ ^]^ Finally, one should mention a remarkable recent example, where Anderson group assembled 24 porphyrins into a ring, which mimics rings of chlorophyll molecules found in purple bacteria of natural LH antenna (**6**, Figure 3).^[^ ^118^ ^]^ Due to close proximity of porphyrins within the ring, the ultrafast excitation energy transfer occurred from the 22 meso‐meso linked porphyrin units to the butadiyne‐linked segment (lower energy) with average time constant of 8.5 ps.

Recently, Würthner and co‐workers introduced heteromeric dye foldamer as a new concept in fabricating LHS.^[^ ^119^ ^]^ It combined four different merocyanine dyes, where peptide‐like foldamer controls co‐facial dye stacking, leading to a panchromatic absorption band consisting of several strongly coupled exciton states. Due to rigid packing with controlled dye‐dye positioning, this LH dye array exhibited high fluorescence QY (38%) and nearly quantitative FRET efficiency (95%).

Overall, these examples of molecular LHS show the power of organic chemistry of dyes reaching this high level of complexity in LHS design. However, here comes also its limitation, as it is difficult to add more donor dyes in these systems, because of synthetic difficulties and increased ACQ of donor dye, which would lead to decreased FRET efficiency.

### 4.2 Aggregates of “Classical” Dyes

**Figure 4:**
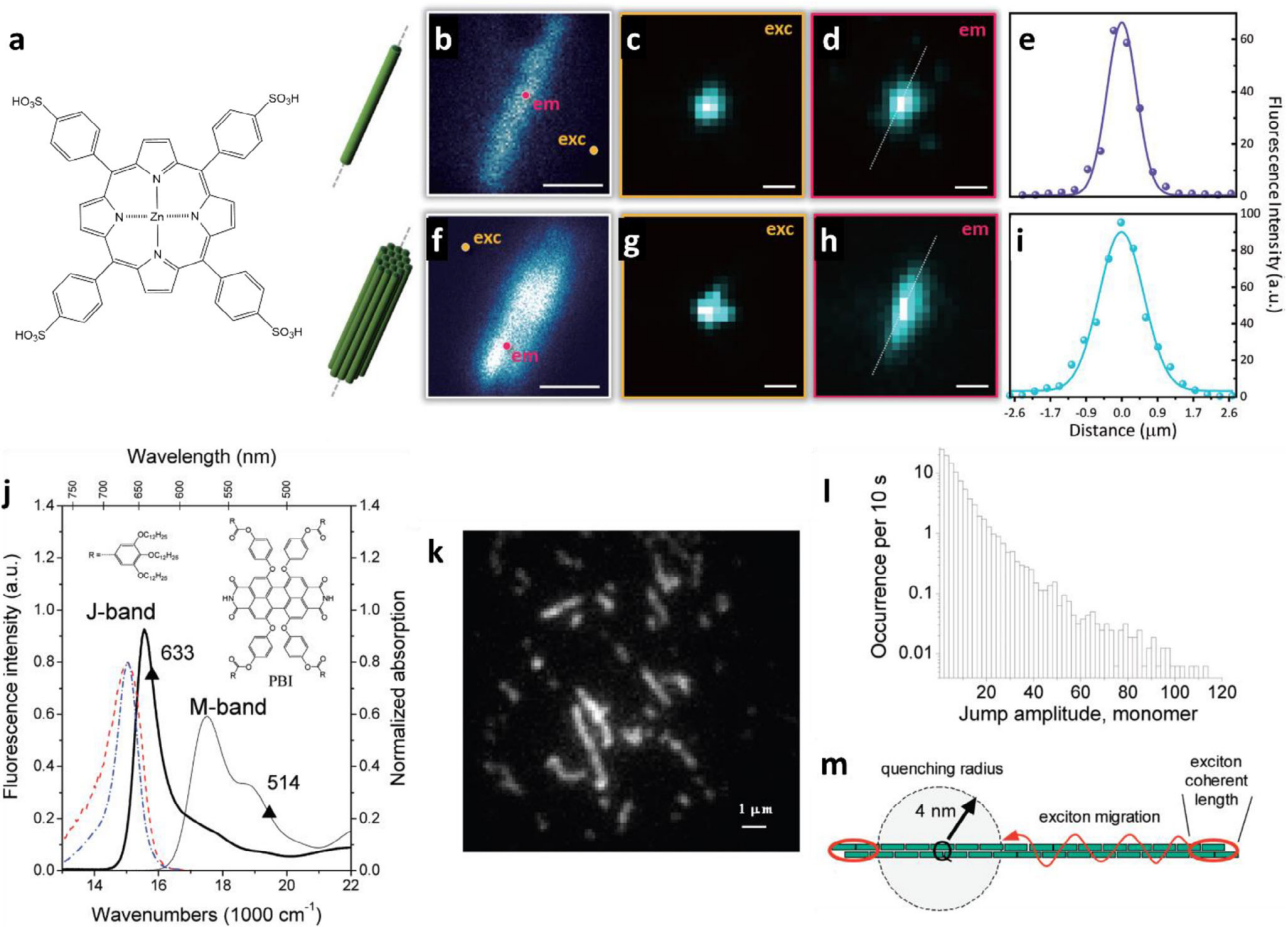
a) Chemical structure of porphyrin derivative TPPS. b and f) Confocal fluorescence scan images of a nanotube and bundle, respectively. c and g) Reflected excitation beam profile after focusing the excitation beam outside the aggregates [on the yellow spots in (b) and (f)]. (d and h) Emission profiles after focusing the excitation beam on selected sites in the aggregates [pink spots in (b) and (f)]. The excitation was performed using a 485 nm diode laser. (e and i) Cross‐section profiles of the emission profiles along the marked white dotted lines. Scale bar = 1 µm in all panels. Reproduced with permission.^[^ ^126^ ^]^ Copyright 2018, the Royal Society. j) Chemical structure of PBI (inset), absorption spectra of PBI monomer solution in DCM (thin solid line) and J‐aggregates in MCH (bold solid line), emission spectrum of the J‐aggregates in MCH solution (dash‐dot line), and typical emission spectrum of an individual J‐aggregate on glass surface (dash line). k) A typical fluorescence image of a sample containing isolated long J‐aggregates with length >1 µm. l) Distribution of fluorescence intensity jump amplitudes in single J‐aggregates of PBI, represented by the average occurrence per 10 s under excitation. m) Schematic illustration of the effect of quenching radius, exciton coherent length, and migration distance on the jump amplitude (number of collectively quenched monomers) in PBI J‐aggregates. Reproduced with permission.^[^ ^35^ ^]^ Copyright 2010, American Chemical Society.

The simple approach in construction of LH system is to combine chromophores within non‐covalent nanoaggregates, which can be in amorphous or crystalline form. We first focus on aggregates of “classical” dyes, which include well‐established fluorophores, while the AIE chromophores are presented in the next chapter. The primary examples are aggregates of porphyrin dyes, which are the closest analogues of natural LHS. The early studies revealed that chlorophyll and other porphyrin dyes tend to assemble into nanorods.^[^ ^120^, ^121^ ^]^ With appropriate molecular design assemblies of porphyrins may exhibit red shifts in absorption with some band broadening as well as detectable fluorescence, suggesting formation of J‐aggregate like structures.^[^ ^122^ ^]^ Remarkably, in these aggregates, organization of synthetic porphyrin analogues can be similar to that in natural LHS.^[^ ^120^, ^123^ ^]^ J‐aggregates of porphyrins typically displayed fast dye‐dye EET at the times scale of ≈1 ps^[^ ^124^ ^]^ and the exciton diffusion lengths on the order of 100 nm.^[^ ^125^ ^]^ Recent advanced optical microscopy studies of J‐aggregates of meso‐tetra(4‐sulfonatophenyl) porphyrin (TPPS, **Figure** **4**a) revealed important role of coherent transfer^[^ ^125^ ^]^ and demonstrated an impressive exciton diffusion length of 183 and 457 nm for nanotubes (1D system) and bundles (3D system), respectively (Figure 4b‐i).^[^ ^126^ ^]^

Würthner and co‐workers coupled porphyrin Zinc chlorin with an energy donor dye naphthalene bisimide (NBI) in a dyad and further assembled them into nanorods.^[^ ^127^, ^128^ ^]^ The obtained structures exhibited quantitative FRET (99%) with ≈5 ps kinetics and extended efficient energy harvesting in the green spectral region, so called “green gap” in natural LHS.^[^ ^127^ ^]^ The same group also developed J‐aggregates based perylene bisimide (PBI) dyes^[^ ^129^ ^]^ J‐aggregates of one of those PBI derivatives showed collective fluorescence blinking, which was measured by wide‐field fluorescence microscopy (Figure 4j–m).^[^ ^35^ ^]^ These results corresponded to collective quenching of up to 100 dyes coupled by EET, with exciton diffusion length up to 70 nm in these 1D assemblies (Figure 4l,m). Here, one should also mention the work of Métivier and co‐workers, where organic nanoparticles were assembled from a push‐pull dye coupled with a photo‐switchable molecule.^[^ ^130^ ^]^ These NPs exhibited remarkably high fluorescence quantum yield (≈60%), and it was shown that only 5% of photo‐conversion could switch emission of the whole particle. Indeed, a single converted photochromic molecule ensured photo‐switching of 420 ± 20 of dyes within the particle. This is a remarkably example of LHS, which provided strong amplification of photo‐switching at the nanoscale. Other examples of potential LHSs include (nano)materials based on push‐pull dyes,^[^ ^131^ ^]^ bulky perylene diimides,^[^ ^57^ ^]^ polycyclic aromatic hydrocarbons co‐crystallized with octafluoronaphthalene,^[^ ^65^ ^]^ crystalline BODIPY‐proline conjugates,^[^ ^132^ ^]^ etc. Despite the presence of multiple examples of LHS based on classical dyes, vast majority of aggregates of classical dyes undergo ACQ, which limits their LHS performance. Therefore, dedicated approaches to prevent ACQ in organic dye‐based materials were elaborated.

### 4.3 Aggregation‐Induced Emission Nanomaterials

Aggregation‐induced emission (AIE) was introduced by Tang and co‐workers in 2001^[^ ^59^ ^]^ with the aim to propose a generic solution against ACQ of organic dyes.^[^ ^60^ ^]^ AIE concept stimulated a remarkable development in the field of artificial LH systems with AIE fluorophores (AIEgens), leading to a broad range of applications, such as sensing,^[^ ^133^, ^134^, ^135^, ^136^, ^137^ ^]^ opto‐electronics,^[^ ^138^, ^139^, ^140^, ^141^ ^]^ biomedical applications^[^ ^142^, ^143^, ^144^, ^145^ ^]^ etc. Here, we will focus on key examples AIE nano‐assemblies as LHS.

Typical AIE dye (AIEgen), like tetraphenylethylene, is poorly emissive in solution, because its molecular rotations and vibrations ensure fast non‐radiative deactivation through an internal conversion from the singlet excited state to the ground state.^[^ ^60^ ^]^ When AIEgens get aggregated, restrictions of intramolecular motions (RIM) suppress their quenching pathway through rotations and vibrations, leading to their efficient emission in the solid state. Multiple related mechanisms of AIE have been also proposed,^[^ ^146^ ^]^ including restriction of vibronic coupling (RVC),^[^ ^147^ ^]^ restriction of access to conical intersection (RACI)^[^ ^148^ ^]^ and restriction of access to dark state (RADS).^[^ ^149^, ^150^ ^]^

Based on these different mechanisms, over last few years, a number of artificial LH systems have been developed. In these systems, energy acceptor molecules are introduced into the assembly of AIEs donors with controlled donor/acceptor ratio, which leads to light harvesting by the AIE dyes followed by efficient energy transfer to the acceptor. These AIE‐based artificial LHS are broadly classified into two different classes: 1) covalent frameworks and 2) non‐covalent frameworks.^27^

**Figure 5:**
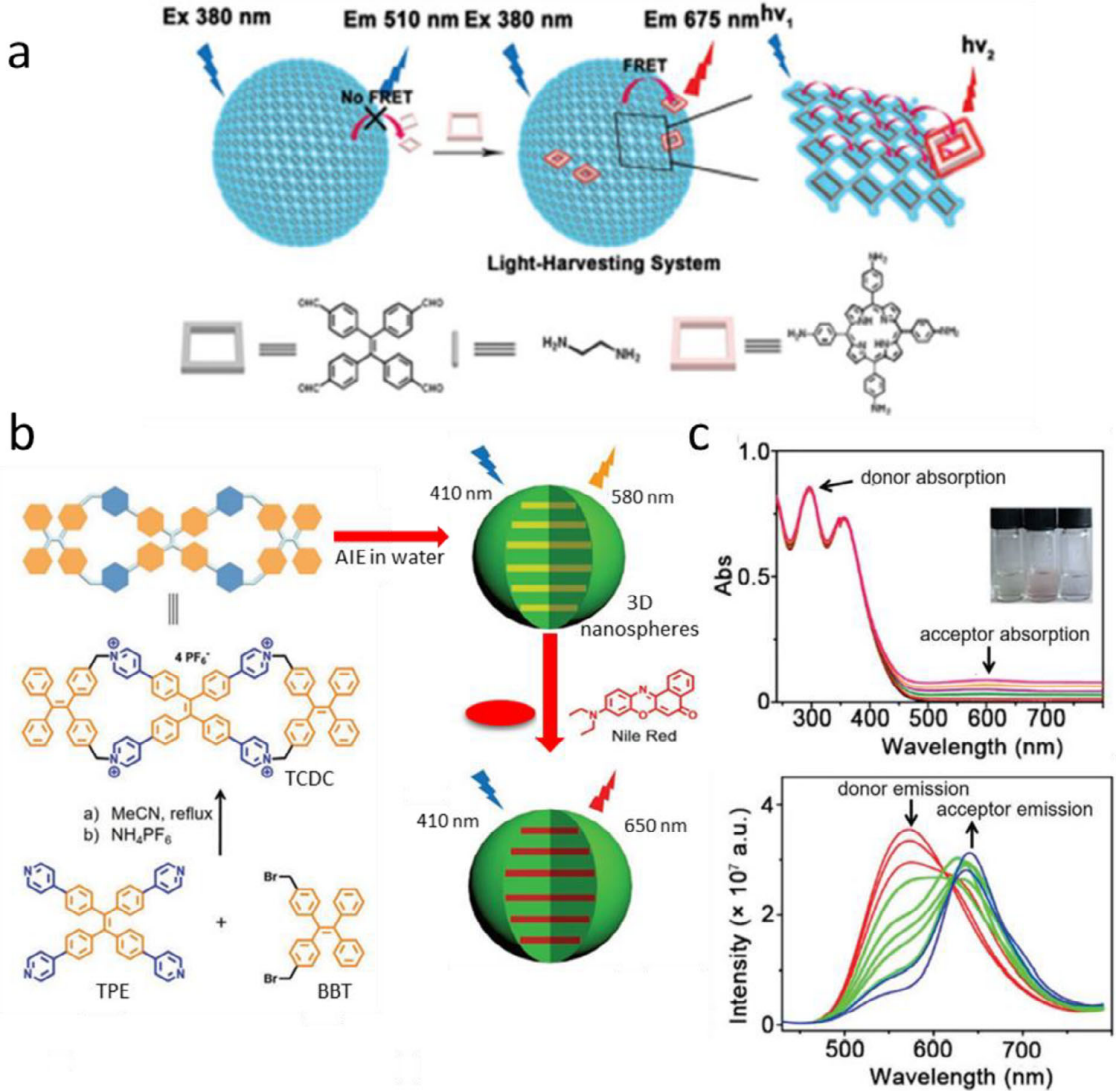
a) The structure of AIE molecules and the proposed formation of 2D polymer and polymer hollow nanosphere. Reproduced with permission.^[^ ^151^ ^]^ Copyright 2019, John Wiley and Sons. b) Illustration of the construction of the light‐harvesting system and the process of light‐harvesting of 3D nanospheres of TCDC AIEgen and Nile Red. c) Absorption and emission spectra of the 3D nanospheres at different donor‐acceptor ratio. Reproduced with permission.^[^ ^152^ ^]^ Copyright 2019, American Chemical Society.

Covalent frameworks ensure high level of structural ordering of chromophores and enhances the stability of the systems. In 2019, Liu and co‐workers have synthesized an AIEgen 4,4′,4″,4′‐(ethene‐1,1,2,2‐tetrayl) tetrabenzaldehyde (TPE), which eventually reacts with ethylenediamine and forms 2D oligomeric patches by Schiff base reaction (**Figure** **5**a).^[^ ^151^ ^]^ In ethanol, these 2D patches transform into nanovesicles due to surface tension. The nanovesicles with size of 510 nm showed higher QY than its non‐aggregated states which confirmed AIE. To form artificial LHS, a porphyrin derivative (TAPP) having good spectral overlap with TPE was used as an acceptor covalently linked to the surface of these nano‐vesicles. Addition of acceptor gradually led to the loss in donor intensity at 510 nm and increase in the acceptor intensity at 675 nm, confirming FRET. The FRET efficiency using the average lifetimes of the donor was 50.4% at 20:1 molar ratio of TPE to TAPP. Thus, an artificial LHS was fabricated that exploited AIEgens in form of nano‐vesicles.

Cao and colleagues synthesized a tetracationic dicyclophane (TCDC, Figure 5b) by reacting tetrapyridyl‐based tetraphenylethene (TPE) and bis(bromomethyl) tetrabenzaldehyde (BBT) in a 1:2 molar ratio.^[^ ^152^ ^]^ Characterization by SEM, TEM, and DLS revealed that TCDC self‐assembles into 3D nanospheres with diameters ranging from 25 to 77 nm (Figure 5b) in acetonitrile or an acetonitrile‐water mixture. By incorporating Nile Red as an acceptor dye within these nanospheres, artificial LHS were obtained. Notably, TPE exhibited a decrease in emission alongside a concurrent increase from Nile Red, indicating efficient energy transfer from TPE to Nile Red (Figure 5c). The antenna effect and energy transfer efficiency were measured at 14.3 and 77.5%, respectively.

**Figure 6:**
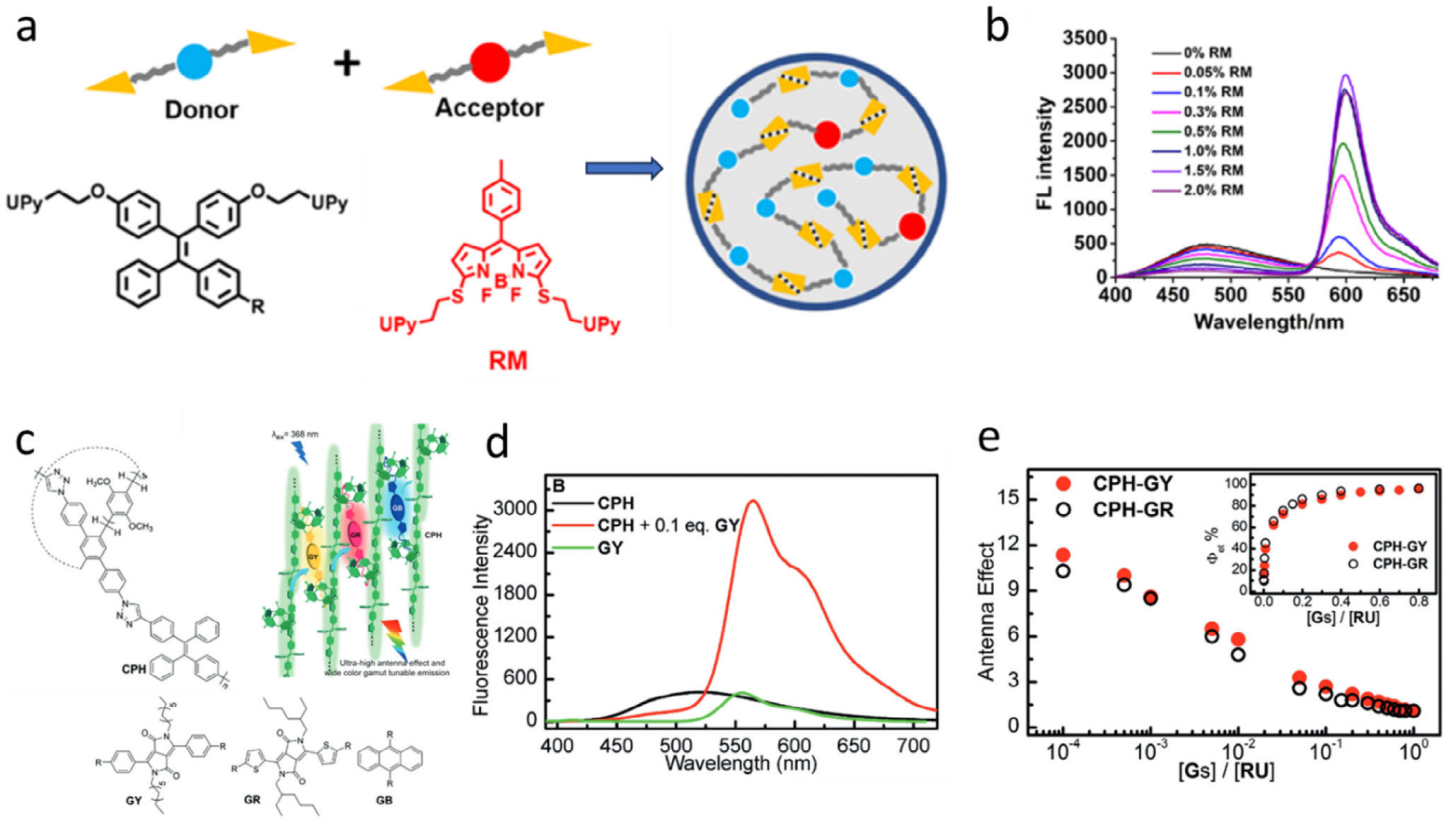
a) Schematic illustration of the preparation of AIE materials with light‐harvesting properties, and chemical structures of the UPy‐modified donors (TPEH) and acceptor (RM). b) Fluorescence spectra of TPEH‐RM with different molar percentages of RM, λ_ex_ = 350 nm. Reproduced with permission.^[^ ^155^ ^]^  Copyright 2019, American Chemical Society. c) Molecular structures for the conjugated polymeric host CPH, and the guests (GB, GY, GR). d) Fluorescence response of CPH toward GY in the co‐solvent of CHCl3/c‐hexane (3:7 by volume). [RU] = 10 µM, [GY] = 1 µM, λ_ex_ = 368 nm. e) Efficiencies evaluated by using AE and Φ_ET_ (insert) in the co‐solvent of CHCl_3_/c‐hexane (3:7 by volume). [RU] = 10 µM. Reproduced with permission.^[^ ^162^ ^]^ Copyright 2019, John Wiley and Sons.

In spite of having important advantages, the covalent framework approaches often tend to have synthetic hassles as well as less product yields. In contrast, nature has always used supramolecular frameworks for LHS,^[^ ^153^ ^]^ which inspired researchers to develop supramolecular LHS.^[^ ^1^, ^154^ ^]^ Recently Yang and co‐workers fabricated a series AIE NPs with LHS features based on supramolecular polymerization using hydrogen bonding interactions.^[^ ^155^ ^]^ The 2‐ureido‐4[1H]‐pyrimidinone (UPy)‐modified molecule has a quadruple hydrogen bonding unit, which is widely used for supramolecular assemblies, because it is known that UPy forms stable supramolecular architectures.^[^ ^156^ ^]^ They used UPy‐modified tetraphenylethene as a donor and UPy‐modified BODIPY as an acceptor (**Figure** **6**a). The nanoparticles were prepared using different ratios of donor and acceptor molecules. With increasing amount of the acceptor, the donor fluorescence intensity decrease was accompanied by the rise of the acceptor fluorescence intensity (Figure 6b). Moreover, the decrease in average lifetime of the donors from 3.65 ns to 1.48 ns upon addition of acceptor molecules confirmed FRET behavior, which corresponded to 60% FRET efficiency. More interestingly, compared to the NPs without acceptor, the NPs with acceptor showed narrow emission band with lowering of full width half maxima (FWHM) and also with 5.7‐fold increased QY (3% without acceptor to 17% with acceptor) within the valuable wavelength range with 6‐fold amplified FRET signal. Moreover, white light fluorescence has been observed by doping only 0.1% of acceptor molecules in pure donor NPs. Taking advantage of low FWHM, the obtained NPs were applied for live cell imaging.

Liu and colleagues developed a supramolecular hyperbranched polymer from a tetraphenylethene derivative containing a pyridinium (Py‐TPE) unit.^[^ ^157^ ^]^ By employing pillar[5]arene (WP5), this Py‐TPE was assembled into 50–200 nm NPs. A negatively charged energy acceptor, sulforhodamine 101 (SR101), was adsorbed on cationic surface of NPs yielding LHS. Under excitation of the donor at 350 nm, fluorescence studies showed a decrease in the emission at 480 nm from the tetraphenylethene derivative and a significant emission increase at 610 nm from SR101, indication of FRET. The FRET efficiency was 64%, with an antenna effect of 13 at a donor‐to‐acceptor ratio of 150:1. These results underscore the role of electrostatic interactions in facilitating effective energy transfer within the artificial LHS.^[^ ^158^ ^]^

Xiao and colleagues developed an efficient artificial LHS, using Upy‐mediated quadruple hydrogen bonding. They synthesized an AIE chromophore (fixed tetraphenylethylene)‐bridged ditopic ureidopyrimidinone (M), capable of self‐assembling into supramolecular polymers. Using a mini‐emulsion method, water‐dispersed NPs of 150 nm size (by TEM) were obtained with emission in water due to the AIE effect with quantum yield of 2.3%. Fluorescence titration of NPs with naphthalenediimide (NDI) dye as acceptor and time‐resolved fluorescence spectroscopy confirmed 58.2% FRET efficiency when only 1% of NDI was co‐assembled with NPs. At an NP‐to‐NDI ratio of 500:1, antenna effect of 16 was observed.^[^ ^159^ ^]^

In a subsequent study, the same group developed a stepwise energy‐transfer LHS using a similar tetraphenylethylene (TPE)‐bridged UPy derivative as the energy donor, with two hydrophobic dyes, DBT and NDI, acting as the first and second energy acceptors, respectively.^[^ ^160^ ^]^ Using a mini‐emulsion approach in aqueous media and with the aid of CTAB, they generated supramolecular polymeric NPs with tunable fluorescence (with a donor QY of 80%) from blue to near‐infrared, including white light emission. This two‐step LHS achieved a high antenna effect, reaching up to 63 with a donor/DBT/NDI ratio of 1250/25/1. This cascade energy‐transfer system based on supramolecular polymeric NPs holds great promise for mimicking photosynthesis and advancing the development of tunable organic fluorescent materials.

Liu and colleagues designed an efficient artificial LHS by encapsulating the hydrophobic dye Nile Red (acceptor) within nanoparticles, that were formed through a straightforward host–guest interaction between sulfato‐β‐cyclodextrin derivative and an oligo(phenylenevinylene) derivative.^[^ ^161^ ^]^ The inclusion of sulfato‐β‐cyclodextrin gave the LHS excellent water solubility, while oligo(phenylenevinylene) derivative (donor) exhibited excellent AIE properties that enhanced energy transfer to Nile Red even when aggregated. With a donor‐to‐acceptor ratio of 125:1, the energy transfer efficiency and antenna effect were calculated to be 72% and 32.5, respectively—exceeding many other artificial LHSs in aqueous solutions.

Jia and colleagues have successfully developed an artificial LHS utilizing β‐cyclodextrin (β‐CD) as a host molecule. In this system, β‐CD is functionalized with naphthalimide (NDI) to serve as the donor molecule, while the guest molecule, adamantane, is modified with tetraphenylethylene (TPE), which exhibits aggregation‐induced emission (AIE) properties.^[^ ^163^ ^]^ The host‐guest interaction between the β‐CD‐modified NDI and the adamantane‐TPE complex facilitates the spontaneous formation of stable, uniformly sized nanoparticles in water. The close proximity and substantial spectral overlap between naphthalimide and tetraphenylethylene enable efficient FRET with 57.0% efficiency and antenna effect of 13.0 at 40:1 donor‐to‐acceptor ratio. This innovative approach offers a promising method for constructing artificial light‐harvesting systems operating in aqueous environments.^[^ ^163^ ^]^

Wang and Hu and colleagues recently developed two efficient artificial LHS with AIE through the self‐assembly of a salicylaldehyde azine, a water‐soluble pillar[6]arene derivative, and fluorescent dyes Nile Red and Eosin Y (ESY) via host–guest interactions with an antenna effect of 28 at 200:1 donor to acceptor ratio.^[^ ^164^ ^]^ They later constructed a two‐step sequential energy transfer LHS by combining pillar[5]arene derivative and a bola‐type tetraphenylethylene‐modified dialkyl ammonium derivative, creating in water a stable complex, which self‐assembled into multilayer nanoparticles with an average diameter of 180 nm.^[^ ^165^ ^]^ The energy transfer from tetraphenylethylene to ESY achieved a 74.39% efficiency and an antenna effect of 11.5 at a donor‐to‐acceptor molar ratio of 200:1. Next, Nile Red was introduced leading a two‐step energy transfer—from blue emitting tetraphenylethylene to ESY, then to red emitting Nile Red.^[^ ^165^ ^]^ The same group also used pillar[5]arene to assemble bola‐type bis(4‐phenyl)acrylonitrile derivative (BPT, donor) with different hydrophobic fluorescent dyes (4,7‐bis(thien‐2‐yl)‐2,1,3‐benzothiadiazole (DBT, first acceptor) and Nile Red (second acceptor).^[^ ^166^ ^]^ At the first FRET step for the donor/acceptor ratio of 350/1, a high antenna effect (47.8) was achieved.

In a recent study, Zhou and co‐workers developed small‐sized AIEdots (18–36 nm in diameter), containing high concentration of AIEgen (TPE‐BT and TPA‐BT) and low concentration of organic dyes (such as BODIPY, cyanine and phthalocyanine derivatives) with narrow emission bands.^[^ ^167^ ^]^ At donor/acceptor ratio 200/1, FRET efficiency of >90% was achieved. These NPs were characterized also by high fluorescence quantum yield (≈70%) and narrow emission, which is important for multiplexed imaging in vitro and in vivo. In another study, Sun and co‐workers developed spirocyclic skeleton bridged tetraphenylethylene dimer, which assembled in water into LH nanoparticles of 150 nm dimeter.^[^ ^168^ ^]^ In the presence of Nile Red as acceptor, the FRET emission was observed. At 250/1 donor/acceptor ratio, the antenna effect reached 66.

Tang and Cao et al, has described a novel strategy for fabricating supramolecular artificial LHSs using AIEgens and through host‐guest interactions (Figure 6c). The authors made this LHS with a conjugated polymeric supramolecular network, which was created from the self‐assembly of a pillar[5]arene‐based conjugated polymeric host (CPH), which contains AIEgens such as tetraphenylethylene moieties (donors) and conjugated ditopic guests such as diketopyrrolopyrrole or anthracene derivatives (acceptors). The guest molecules were bound to the host cavity, which eventually prevented the ACQ ensuring EET. The antenna effect of 35.9 was measured in solution of c‐hexane for this system, whereas they found antenna effect value of 90.4 in solid state for the donor/acceptor ratio of 1000:1 (Figure 6d,e).^[^ ^162^ ^]^

Overall, the AIE concepts enabled for fabricating efficient LHS using both covalent framework approach and non‐covalent approach. Nevertheless, the efficiency of AIE LHS is limited by two factors: relatively small molar extinction coefficient and large Stokes shift of AIE dyes. Indeed, the twisted structure is important for high QY of the AIE systems in the solid state, but it also decreases the π‐conjugation and thus reduces the extinction coefficient, which is directly linked to LH properties. Second, large Stokes shift decreases the donor‐donor spectral overlap and thus slows done EET. Therefore, further efforts are currently going on to increase the planarity of the system and to increase the LH antenna amplification effect.

### 4.4 Metal–Organic Frameworks

The dyes can be assembled into LHS due to metal coordination. The early examples, reviewed by Würthner and co‐workers,^[^ ^2^ ^]^ included preparation of molecular LH arrays of multiple chromophores connected together by coordination bonds with metal ions. They present similar features as covalent molecular LHS described earlier, and will not be reviewed here. To go beyond molecular systems into mesoscale using ion coordination, metal‐organic frameworks (MOFs) were introduced.^[^ ^169^, ^170^, ^171^, ^172^, ^173^, ^174^ ^]^ The exponential growth of research in MOFs reflects their immense potential across diverse applications, including catalysis, separations, gas storage, drug delivery, optics and magnetism.^[^ ^169^, ^170^, ^171^, ^172^, ^173^, ^174^ ^]^ Beyond these functional roles, MOFs have recently emerged as promising candidates as light‐harvesting materials due to their unique structural attributes.^[^ ^175^, ^176^, ^177^ ^]^ MOFs consist of organic struts connecting “nodes,” which can be metal ions (either single or paired), metal‐oxy nanoclusters, or related constructs. MOFs exhibit a compact architecture, analogous to natural photosynthetic antennae that rely on chromophore arrays for light harvesting.^[^ ^75^, ^80^ ^]^ However, unlike natural systems, MOFs generally display relatively weak chromophore coupling, making FRET the dominant mechanism for energy transfer in these systems.^[^ ^178^ ^]^ This reliance on FRET underlines the importance of carefully designing MOF structures to optimize energy transfer efficiency, which is a key requirement for light‐harvesting applications. The porosity of MOFs ensures intermolecular spatial separations between chromophores, circumventing the effects of ACQ, which is detrimental to LHSs. Additionally, the crystallinity of MOFs provides structural rigidity and tunability, facilitating energy transport by bandgap modulation or tuning electronic overlaps between donors and acceptors.

Energy transfer processes in MOF‐based nanomaterials can be broadly categorized into four types:^[^ ^179^ ^]^ 1) ligand‐to‐ligand energy transfer (ET1): Energy is transferred between chromophoric ligands within the MOF structure; 2) metal‐to‐metal energy transfer (ET2): Energy is exchanged between metal centers in the framework;^[^ ^180^, ^181^ ^]^ 3) ligand‐to‐metal or metal‐to‐ligand energy transfer (ET3): Energy transfer occurs between the organic ligands and the metal centers;^[^ ^182^, ^183^ ^]^ 4) MOF‐to‐guest or guest‐to‐MOF energy transfer (ET4): Energy is transferred between the MOF framework and encapsulated or surface‐bound guest molecules. This review primarily focuses on ET1 and ET4 mechanisms, while mechanisms ET2 and ET3 remain outside the scope of this discussion but represent intriguing avenues for future exploration.

**Figure 7:**
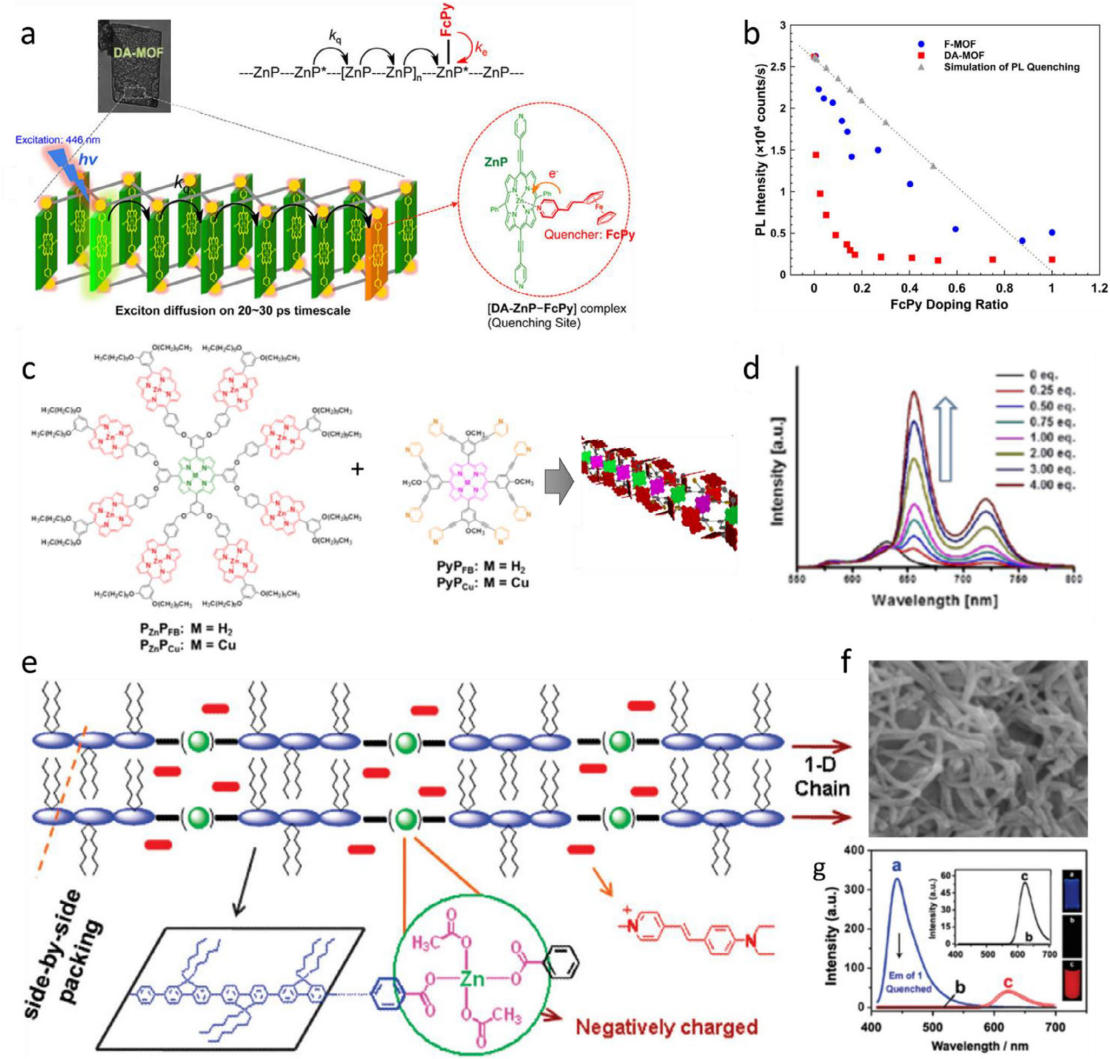
a) Photograph of a DA‐MOF particle from which fluorescence is recorded based on laser excitation at 446 nm. Schematic representation of the exciton migration and quenching processes. Upon illumination, the photogenerated exciton can hop between adjacent porphyrin units until it reaches a quenching site. In the red ellipse is an expanded view of the quenching site where the quencher (FcPy) is bound to the porphyrinic Zn center; the exciton is quenched via electron transfer from FcPy to the porphyrin entity (ZnP). b) Steady‐state fluorescence intensities of F‐MOF (closed blue circles) and DA‐MOF (closed red squares) as a function of extent of FcPy loading (from 0 to 1). The linear gray plot shows the behavior expected when exciton migration (EET) is absent and quenching is exceptionally fast relative to the exciton lifetime. Reproduced with permission.^[^ ^180^ ^]^ Copyright 2013, American Chemical Society. c) Chemical structures of multiporphyrin dendrimers (P_Zn_P_M_) and multipyridyl porphyrins (PyP_M_) and schematic illustration of supramolecular polymers. d) emission changes of P_Zn_P_Cu_ by successive addition of PyP_FB_; (d) emission changes of P_Zn_P_FB_ by successive addition of PyP_Cu_. All emission spectra were measured in toluene at 25 °C. Reproduced with permission.^[^ ^185^ ^]^ Copyright 2015, American Chemical Society. e,f) Proposed self‐assembly mechanism for 1‐D nanostructures (e) and their SEM image (f). (g) The emission spectra of 1 (curve a), 1 + 2 (curve b), and 1 + 2 + Zn(OAc)_2_ (curve c) in DMF. All of the spectra were obtained by excitation at 370 nm. The inset shows fluorescence images under 365‐nm UV illumination and expansion of the curves b and c. Reproduced with permission.^[^ ^186^ ^]^ Copyright 2009, American Chemical Society.

MOFs provide a unique structural platform for *ligand‐to‐ligand energy transfer*, which can use different ligands as energy donors and acceptors, facilitating efficient FRET within the framework. Pioneering work of Hupp and co‐workers exemplifies this approach through the development of a FRET‐based MOF system known as BOP‐MOF.^[^ ^184^ ^]^ This system employed a pillared paddlewheel architecture, utilizing pyridine‐functionalized BODIPY struts as donor ligands and zinc‐porphyrin derivatives as acceptors. The donor ligands form the pillars of the framework, while the zinc‐porphyrin‐based acceptors comprise the paddlewheels in a Zn(II)‐based MOF structure. Upon excitation of the BODIPY pillars at 543 nm in DMF, energy transfer to the porphyrin paddlewheels results in characteristic emission at 667 nm, clearly demonstrating the occurrence of FRET. The study further highlights the efficiency of energy transfer by calculating the exciton diffusion length within the MOF. Using a ferrocene‐based quencher, the group determined that excitons diffuse over an average distance of 45 porphyrins within their lifetime (**Figure** **7**a,b). This corresponds to a net exciton migration distance of 58 nm with nearly 100% energy transfer efficiency.^[^ ^180^, ^184^ ^]^

In another report, Lee et al. described a 1D supramolecular coordination polymer of multiporphyrin dendrimers (donor) (P_Zn_P_M_; where M = freebase (FB) porphyrin or Cu) as an artificial LHS. The P_Zn_P_M_ is composed of a central porphyrin with Cu or FB with eight zinc porphyrines (P_Zn_) at the terminal.^[^ ^185^ ^]^ They also synthesized multipyridyl porphyrin acceptors (P_Y_P_M_; where M = FB or Cu) (Figure 7c,d), bearing eight pyridyl groups, suitable to form coordination bonds with P_Zn_. By differential absorption spectroscopy they found that the P_Y_P_FB_ is forming 1:1 complex with P_Zn_P_FB_ in toluene. AFM and TEM suggested that this complex assembles into nanofibers of 6 nm diameter in toluene. They demonstrated an efficient intramolecular energy transfer from the P_Zn_ wings to P_FB_ of P_Zn_P_FB_. When excited at 414 nm (P_Zn_ wings) in toluene, the system exhibited strong emission bands at 650 and 720 nm from the focal P_FB_, along with a faint shoulder at 600 nm from the P_Zn_ wings, indicating efficient energy transfer (94%). However, when P_Zn_P_FB_ was mixed with P_y_P_FB_, the shoulder at 600 nm disappeared entirely, and the emission bands at 650 and 720 nm shifted slightly to longer wavelengths. The shifted emissions matched the emission spectrum of P_y_P_FB_, suggesting that the energy is being transferred intermolecularly to P_y_P_FB_. To confirm this, researchers prepared non‐fluorescent Cu‐porphyrin complexes. When P_y_P_FB_ was added to the non‐fluorescent P_Zn_P_Cu_, the complex became fluorescent. This indicates that the energy from the P_Zn_ wings is no longer being transferred to the focal P_Cu_ but is instead transferred to P_y_P_FB_.^[^ ^185^ ^]^ Overall, this study presents an effective approach for controlling and tuning energy transfer pathways, both within a molecule and between molecules.

Sun and coworkers demonstrated this advantage through a “4+4 strategy” to develop a MOF‐based LH antenna complex.^[^ ^187^ ^]^ They synthesized a sodalite like zeolitic MOF based on (Et_2_NH_2_)[In(BCBAIP)].4DEF.4EtOH through the 4 + 4 synthetic strategy by cross‐linking the designed four‐connected tetracarboxylate ligand H_4_BCBAIP with the four‐connected In(III) ion in MeOH as a solvent.^[^ ^187^ ^]^ By carefully selecting a guest acceptor molecule, such as coumarin 6 (loading 0.087 wt%) and coumarin 343 (loading 0.072 wt%), and maximizing the spectral overlap between donor and acceptor components, their design allowed for >35% energy transfer efficiency from the MOF framework to the guest molecule, underscoring the potential of MOFs in light‐harvesting applications.

Zhang et al. fabricated an artificial LHS using nanofibers made up of Zn(II) tuned assembly of a fluorene based ligand, which acted as donor, and trans‐4‐[4 ‐(N,Ndiethylamino)styryl]‐N‐methyl pyridinium ion, which was used as acceptor.^[^ ^186^ ^]^ Mixing of the donor ligand with zinc acetate and the acceptor molecule in DMF (ratio 1:1:2) in DMF, resulted in the quenching of the donor emission (440 nm) with significant increase in acceptor emission (620 nm), indictor of FRET, whereas FRET signal was not detectable without Zn(II) ions. MOFs were obtained in form of nanofibers of 20–30 nm diameter, thus favoring FRET, while their negative charge led to acceptor accumulation and light up inside the nanofibers (Figure 7e,f).^[^ ^186^ ^]^

Alledorf and coworkers developed a novel organic photovoltaic material by integrating MOF‐177 (ZnO_4_(BTB)_2_; BTB = 1,3,5‐benzenetribenzoate) with α,ω‐dihexylsexithiophene (DH6T) and [6,6]‐phenyl‐C₆₁‐butyric acid methyl ester (PCBM) in a solid state and DMF.^[^ ^188^ ^]^ This combination exploited the porous architecture of MOF‐177 to facilitate efficient cascade energy transfer in MOF‐177 from DH6T to PCMB molecules. The precise arrangement and spectral overlap achieved within this MOF resulted in an efficient energy transfer (88%). This study highlights the potential of MOFs as platforms for enhancing the performance of hybrid organic photovoltaic systems.

Even though MOF appears as powerful systems with superior structural control of dye organization, the research direction on light‐harvesting MOF and energy transfer is still underexplored and lacks systematic data on LH nanoantenna performance. Another important challenge, especially for biological applications of LH systems based on MOF is their stability and dispensability in water. In this respect, water‐resistant MOFs based on Zr cation^[^ ^189^, ^190^, ^191^, ^192^, ^193^ ^]^ constitute a fruitful research direction to explore.

### 4.5 Ion‐Associated Nanomaterials

**Figure 8:**
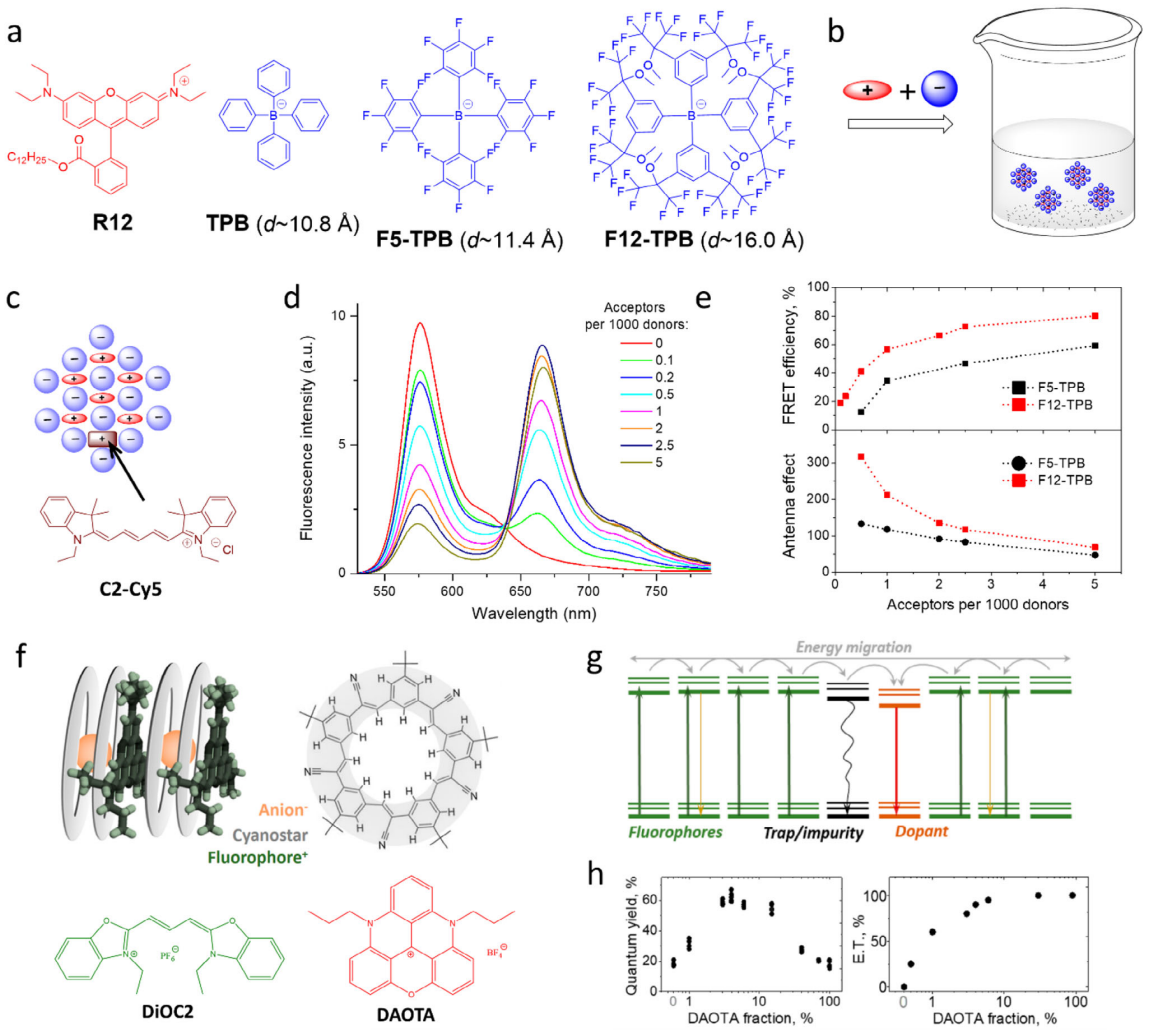
a) Chemical structures of the rhodamine B derivatives (R12) and the different tetraphenylborate counterions used. b) Scheme of self‐assembly of NPs by ion association of cationic dye with bulky hydrophobic counterion. c) Chemical structure of the C2‐Cy5 acceptor and its encapsulation into counterion‐assembled NPs. d) Fluorescence spectra of R12 NPs with F12‐TPB counterion containing different amounts of C2‐Cy5 acceptor dye showing FRET from R12 to C2‐Cy5. Excitation wavelength was 520 nm. e, top) FRET efficiency calculated from the donor fluorescence for NPs with F5‐TPB and F12‐TPB counterions at different acceptor concentrations. e, bottom) Antenna effect calculated from the excitation spectra (ratio of donor intensity maximum to the acceptor intensity maximum emission was detected at 680 nm) for NPs with two different counterions. Reproduced with permission.^[^ ^199^ ^]^ Copyright 2015, the Royal Society. f) Schematic representation of the molecular packing structure in SMILES materials and structure of the CS macrocycle and donor (DiOC2) and acceptor (DAOTA) dyes used. g) State diagram representing FRET‐based SMILES crystals. h, left) Changes in the quantum yield as a function of DAOTA doping. h, right) Energy transfer efficiency in DiOC2‐DAOTA FRET SMILES crystals as a function of DAOTA doping. Reproduced with permission.^[^ ^200^ ^]^ Copyright 2022, American Chemical Society.

The most common and vibrant families of dyes are cyanines and rhodamines.^[^ ^194^ ^]^ However, both of these types of dyes are ionic in nature and tend to aggregate in confined spaces or in aqueous media due to their large flat π‐conjugated structure.^[^ ^195^, ^196^ ^]^ The problem can be addressed by using hydrophobic bulky counterions to control ACQ in a confined space with multiple dyes. These counterions can act as insulators and spacing agents for charged organic fluorophores (**Figure** **8**a,b).^[^ ^23^, ^36^, ^63^ ^]^ Previous research by Yao et al. and Warner et al. has successfully shown that bulky counterions such as tetraphenyl borate (TPB), the fluorinated analogue of TPB (FTPB), bis(2‐ethylhexyl)sulfosuccinate (AOT), and bis(trifluoromethanesulfonyl)imide can increase the QY of the system and help reducing ACQ.^[^ ^47^, ^62^, ^197^, ^198^ ^]^ Our group has further developed this concept by synthesizing a library of organic dye‐based salts with cationic lipophilic rhodamine B (R12) and different hydrophobic bulky counterions (Figure 8a,b).^[^ ^199^ ^]^ All these dye‐counterion pairs were used to fabricate organic dye nanoaggregates. The resulting NPs formed by dye plus bulky hydrophobic ions were around 20 nm in size with high quantum yields such as 60% for F12‐TPB counterion. The counterions with higher fluorination level showed less ACQ and thus higher QY of the NPs. These nanoclusters composed of R12 donors with F12‐TPB counterion and Cy5 derivative as an acceptor at 1000:1 D:A ratio showed >50% FRET efficiency and antenna effect values as high as 200 (Figure 8c‐e). These NPs were 20 times brighter than QD‐585 at 552 nm excitation. Later on, the bulky counterion concept was extended to different polymeric NPs and dyes.^[^ ^23^, ^29^, ^63^, ^64^ ^]^

Flood, Laursen and co‐workers, suggested a crystalline material based on cationic dyes in ionic lattices, named as small‐molecule ionic isolation lattices (SMILES).^[^ ^201^ ^]^ They conceptualized a supramolecular counterion‐based lattice system built by small inorganic ions like BF_4_^‐^, PF_6_^‐^ and ClO_4_^‐^ complexed within π‐conjugated macrocycle, cyanostar (CS). Two CSs caged one small anion by sandwich manner (2+1) generating supramolecular bulky counterion. The latter stacked within cationic dyes in alternating fashion forming a crystal where dye‐dye separation was ≈1.5 nm. This spatial and electronic separation helped to reduce ACQ through H‐aggregation. This concept was successfully applied to variety of dye classes such as rhodamines and cyanines in form of bulk materials. Using intrinsic capacity of CS unit to absorb UV light, LHS based on SMILES were developed that undergo energy transfer from CS to rhodamine or cyanine.^[^ ^202^ ^]^ The materials were obtained in form of NPs stabilized PEGylated lipid with QY of about 30%. In these SMILES NPs, the emission was enhanced by CS excitation with 80% and ≈100% energy transfer efficiency to rhodamine and cyanine, respectively. This UV LH can be attributed by sub picosecond range energy transfer, showing that SMILES NPs constitute a promising material for LHSs. Then, the same groups made prepared SMILES where green‐emitting DiOC2 SMILES crystal (donor) was doped with acceptor dyes, rhodamine 3B (Rh3B) or diazaoxatriangulenium (DAOTA) (Figure 8f–h). At ≈10% fraction of dopant the obtained materials displayed nearly 100% FRET efficiency, suggesting that the crystal showed LHS properties. Moreover, FRET decreased non‐radiative losses in the donor dyes, allowing development of exceptionally bright FRET materials with volume normalized brightness reaching 32 200 M^−1^ cm^−1^ nm^−3^.^[^ ^200^ ^]^ For the volume‐normalized brightness, the fluorescence brightness of a nanomaterial is divided by its volume, which allows evaluation and comparison of brightness of different materials independent of their size.^[^ ^23^ ^]^ High brightness and excellent control of dye‐dye spacing makes SMILES a powerful material for designing artificial LHS.

A recent example of ion‐associated materials, reported by Banerjee and coworkers, explored anionic polymer heparin that co‐assembled with dicationic cyanostilbenes in form of nanotubes.^[^ ^203^ ^]^ Cyanostilbenes served as donors in the light‐harvesting nanoantenna, while Nile Red or Nile Blue was used as acceptors. At donor/acceptor ratios 500 : 1–100 : 1, efficient FRET up to 80% was achieved, which resulted in antenna effects reaching 150. These systems were highly sensitive to temperature, which allowed their application for temperature sensing in the range from 20 till 90 °C.

### 4.6 Hybrid Dye‐Based Systems

#### 4.6.1 Dye‐Biomolecule Hybrids

**Figure 9:**
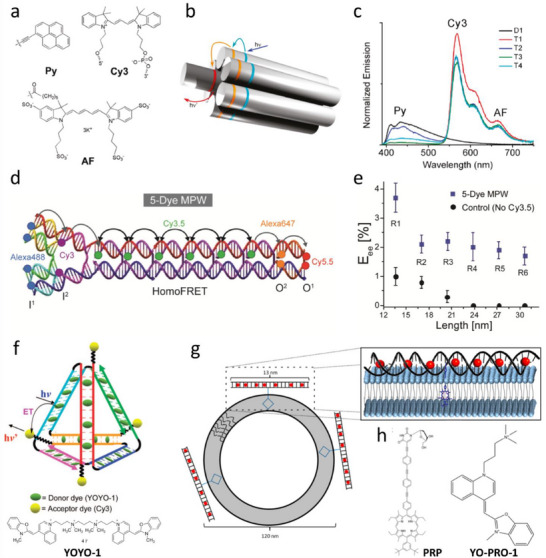
DNA templates for construction of LHS. a) Chemical structure of the chromophores used. b) Schematic display of the self‐assembled 7‐helix bundle (7HB) nanoscaffold that contains three distinct arrays of chromophores: the primary donors, the intermediate donors, and the acceptor, represented by the cyan, orange, and red rings, respectively. c) Normalized emission spectra of D1 and T1‐T4 constructs, which differ by the ratios Py, Cy3 and AF dyes as follows: T1 – Py:Cy3:AF = 6:6:1; T2 – Py:Cy3:AF = 6:3:1; T3 – Py:Cy3:AF = 3:6:1; T4 – Py:Cy3:AF = 1:1:1; D1 – Py:Cy3:AF = 6:0:0. The normalization was done by dividing the emission spectra by the absorption value of each individual sample at 380 nm. Reproduced with permission.^[^ ^209^ ^]^ Copyright 2011, American Chemical Society. d) Schematic of the 5‐dye molecular photonic wires (MPWs). The colored circles represent the dyes, while each DNA strand is presented by a different color. Arrows represent possible energy transfer steps and their directionality. Dye positions are indicated below the MPW and apply to both systems. I = Input and O = Output. e) End‐to‐end efficiency of the 5‐dye MPW as a function of the length of the construct. Error bars are sample deviations from multiple measurements (N > 7). Reproduced with permission.^[^ ^214^ ^]^ Copyright 2016, John Wiley and Sons. f) Schematic description of ET in a tetrahedron nanotag loaded with YOYO‐1 intercalated dyes and covalently attached Cy3 acceptor dyes. Reproduced with permission.^[^ ^221^ ^]^ Copyright 2009, American Chemical Society. g) Schematic representation of the YO−DNA−porphyrin system bound to lipid vesicles (not to scale). A close‐up showing a single DNA−porphyrin construct with seven intercalated YO molecules, bound to a vesicle bilayer via porphyrin. h) Chemical structure of the porphyrin−thymine nucleoside (acceptor). Chemical structure of YO‐PRO‐1 energy donor. Reproduced with permission.^[^ ^97^ ^]^ Copyright 2013, American Chemical Society.

Dye‐biomolecule conjugates are powerful scaffolds for preparation of LHS, because, similarly to natural LHS, biomolecules can provide high control on the dye‐dye spacing and donor‐acceptor composition.^[^ ^204^, ^205^, ^206^ ^]^ DNA and peptides/proteins were the two most popular types of biomolecules explored for LHS preparation. Integration of fluorescent dyes to DNA usually relies on the covalent attachments of the dyes to 5′‐ or 3′‐ends or inside the backbone of the DNA.^[^ ^207^ ^]^ The dye‐DNA conjugates have been widely used in dsDNA,^[^ ^10^, ^53^, ^72^, ^161^, ^162^, ^163^, ^208^, ^209^ ^]^ DNA bricks^[^ ^210^ ^]^ and DNA origami.^[^ ^211^, ^212^, ^213^ ^]^ DNA assembly can help in stoichiometric control and spatial orientations and positions in multi‐chromophore systems. Thus, assembling dyes in DNA origami enables the cascade energy transfer to acceptor. Mimicking natural LH antenna complex, DNA‐based antenna complex was synthesized with self‐assembled seven helix DNA bundles with controlled positioning of pyrene (Py) and cyanine (Cy3) and Alexa Fluor 647 (AF) dyes, forming a circular array of primary and secondary donors and acceptors, respectively. In the complex 6:6:1, the FRET efficiency reached 90% between Py and Cy3, although the measured antenna effect was only 0.85 and 0.93 when emission excited at Py and Cy3, respectively, was compared to that of AF647 (**Figure** **9**a–c).^[^ ^209^ ^]^ Moreover, Medinz and co‐workers studied DNA origami in form of a wire presenting multiple repeats of cyanine dyes as intermediate donors to extend the distance of energy transfer.^[^ ^214^ ^]^ Efficient FRET was observed for both three dye (Alexa488, Cy3 and Cy3.5) and five dye (Alaxa488, Cy3, Cy3.5, Alexa647 and Cy5.5) cascade FRET system (Figure 9d). These complexes displayed stepwise funneling of energy within up to 6 repeats of the same dye, which facilitates the long‐range energy transfer across a distance up to 30 nm (Figure 9e).^[^ ^214^ ^]^ Similar configurations were reported to extend FRET distance length in branched DNA networks^[^ ^215^ ^]^ and three‐layered DNA origami block, allowing FRET over 16 nm distance.^[^ ^216^ ^]^ Dyes were also reported to form J‐ or H‐aggregates via co‐assembly with the dsDNA scaffold, which is important for controlling energy transfer in LHS.^[^ ^217^ ^]^ Recently, pseudocyanine dye was shown to selectively form J‐aggregates on ds‐DNA,^[^ ^218^ ^]^ which present efficient EET within these dyes allowing long‐distance energy transfer between donor and acceptor dyes. Moreover, another cyanine derivative has also been described to show J‐like aggregates in a DNA duplex. It served as a secondary donor with efficient EET within these dyes, which helped to transfer energy from the primary donor to the acceptor to up to 32 nm distance).^[^ ^219^ ^]^ All these DNA templated excitonic systems pave the path to development of new artificial LHSs. However, these examples explored efficient EET in the intermediate donors in order to achieve long distance FRET, whereas the antenna effect in these systems was not expected to be high as the ratio of the primary donor to the acceptor was set to 1–2.

To enhance the number of donors in DNA‐based LHS, dye intercalation strategy could be used. In the early work by Armitage and co‐workers designed a self‐assembled DNA triangle^[^ ^220^ ^]^ and tetrahedron,^[^ ^221^ ^]^ which contained a large number of intercalated dyes. The tetrahedron version showed extinction coefficient of 1.8 × 10^6^ M^−1^ cm^−1^ corresponding to ≈10 YOYO‐1 dyes per tetrahedron (Figure 9f). Remarkably, just a single Cy3 acceptor led to 52% FRET efficiency in the tetrahedron configuration,^[^ ^221^ ^]^ so that the estimated antenna effect (by Equation 10) was ≈6.0. Albinsson and co‐workers reported an artificial LH antenna using intercalation of dyes in DNA (Figure 9g,h). They fabricated a 39‐mer linear DNA duplex containing intercalated donor dye YO‐PRO1 and a porphyrin (PRP) attached at the middle of it and inserted into the lipid bilayer.^[^ ^97^ ^]^ It was found that the antenna effect was 12 when the donor excitation intensity was divided by the excitation intensity of the acceptor. They explained the high efficiency of this system by the homo‐FRET in donors, which allows the energy to migrate through the wire and funnels the whole exciton energy to the porphyrin acceptor. More recently, Häner and co‐workers assembled supramolecular polymers using phenanthrene oligomers (donor) together with oligonucleotides labelled with acceptor (Cy3) dyes.^[^ ^222^ ^]^ At 33/1 donor (phenanthrene trimer) to acceptor ratio, the FRET efficiency of 43% was achieved with antenna effect reaching 22.8. Overall, these works show that DNA is a powerful scaffold that can arrange the chromophores in a nano‐assembly and eventually create an efficient artificial LH antenna complex.

LHS can be also built based on proteins or peptides, which could be considered as closest analogues of natural LHS. An important example is based on dye‐labelled monomers of tobacco mosaic virus coat protein (TMVP). Here, Miller et al. has reported the LHS assembled from monomers modified with donor and acceptor dyes, and achieved high FRET efficiency from multiple donors to a single acceptor. They estimated the antenna effect value of 11.2. Authors suggested that the energy transfer occurred through multiple donor to donor transfer reflecting the increase in antenna effect by increasing the donors to acceptor ratio.^[^ ^223^ ^]^ Other examples of protein‐based artificial LHS are described in the next chapter on micellar systems. One should note that phycoerythrin, allophycocyanin and phycocyanin are typical proteins based LHS, which originate from bacterial LHS. Even though they are an attractive building block for artificial LHS (see below), they mainly known as bright fluorescent labels for imaging and flow cytometry applications.^[^ ^224^, ^225^ ^]^ An original approach proposed by Perrier and co‐workers is to conjugate dyes with cyclic peptides and further assemble them into supramolecular nanotubes.^[^ ^226^ ^]^ The approach allowed aligning the hydrophobic chromophores along the nanotubes in a slipped manner, leading to nanostructures with high fluorescent QY (30%) and a two‐step energy transfer within three different dyes with 95% efficiency. In a two‐component system with a donor/acceptor ratio (pyrene to naphthalene monoimide) of 100/4, the FRET efficiency was 72.6% and antenna effect reached 15.8. Recently, this concept, coined as supra‐fluorophores was generalized to a variety of other dyes, leading to bright fluorescent nanomaterials,^[^ ^227^ ^]^ which will be of interest for fabrication of next‐generation LHS.

#### 4.6.2 Micelles and Supramolecular Polymer Systems

**Figure 10:**
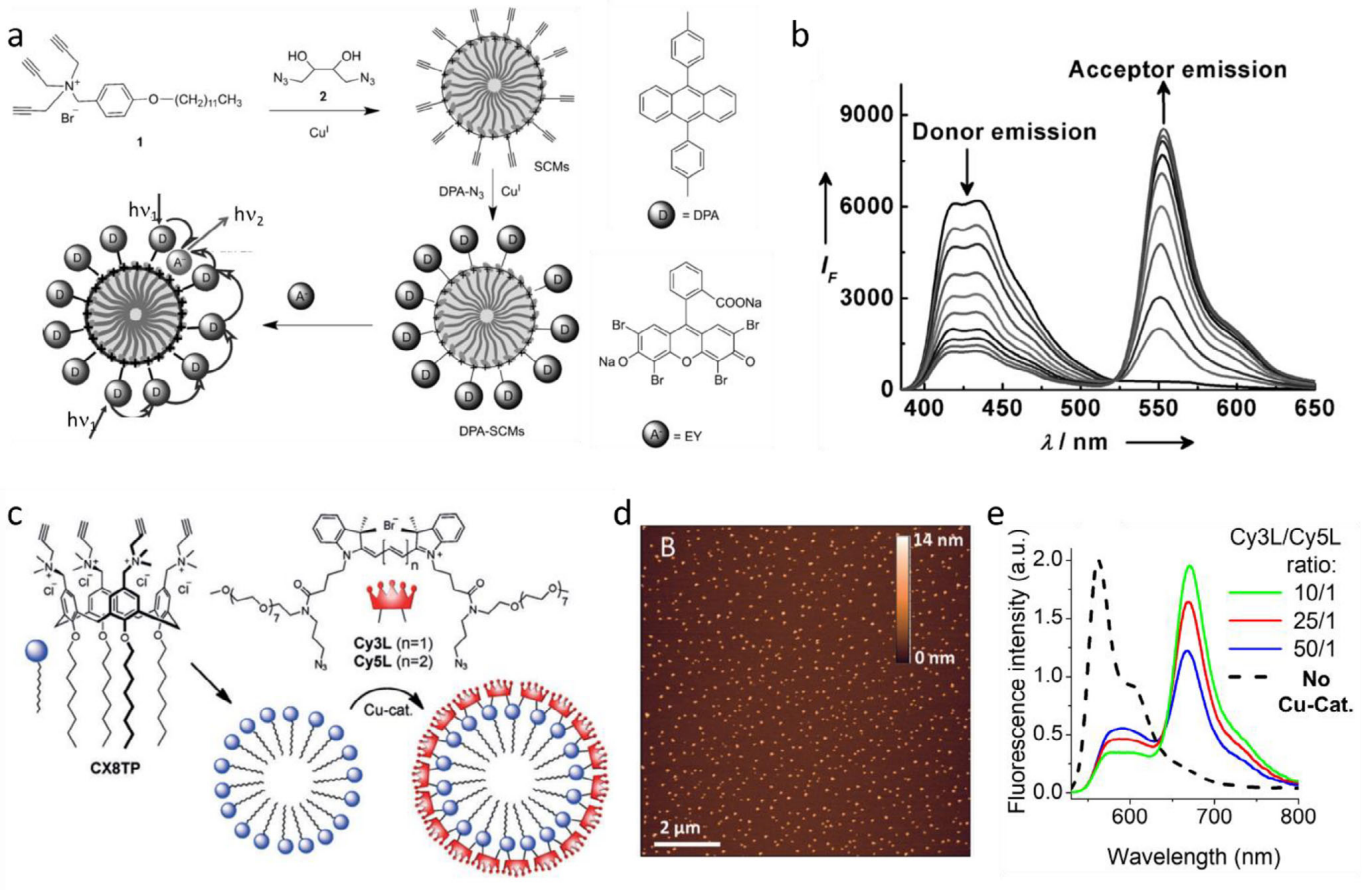
a) Preparation of DPA‐functionalized surface cross‐linked micelles (SCM) and the construction of a light‐harvesting system by introducing EY dye through electrostatic interactions. b) Fluorescence spectra of DPA–SCM in THF with different concentrations of EY. DPA concentration in SCMs was 23.0 mM. EY concentration was 0.00, 0.08, 0.17, 0.33, 0.50, 0.67, 0.84, 1.00, 1.17, and 1.34 mM from bottom to top. λ_*ex*_=375 nm. Reproduced with permission.^[^ ^228^ ^]^ Copyright 2012, John Wiley and Sons. c) Concept of shell‐crosslinking of calixarene micelles with cyanine corona. d) AFM images of shell‐cross‐linked CX/Cy3L micelles. e) Fluorescence spectra of FRET micelles prepared at different Cy3L (donor) to Cy5L (acceptor) ratios in water; a control without cross‐linking in the absence of Cu‐catalyst (no Cu‐Cat.). Reproduced with permission.^[^ ^229^ ^]^ Copyright 2016, John Wiley and Sons.

Nature relies on different covalent and non‐covalent interactions for LH. Covalent interactions provide stability whereas non‐covalent interactions allow assembly of more complex supramolecular structures. For LHS, the basic idea lies on the efficient energy transfer to acceptor from multiple donors for that there is a need to keep a lot of donors close to acceptor molecules. Micelles, nanodroplets and vesicles are attractive material for building artificial LHS. Tong and co‐workers developed an artificial LHS based on surface cross‐linked micelles of a cationic surfactant (1) crosslinked at the surface with a diazide linker (**Figure** **10**a).^[^ ^228^ ^]^ In this antenna system of 25 nm diameter, energy donor 9,10‐bis(4‐methylphenyl)anthracene (DPA) was grafted to the surface by click reaction. The QY of DPA‐micelles reached 90%, which was close to that of free DPA, suggesting the EET from donor to donor without significant energy losses. Eosin Y disodium salt (EY) used as an acceptor was bound to the micelles due to electrostatic interaction, which increased the acceptor emission peak at 550 nm with gradual quenching of the donor emission at 430 nm (Figure 10b). Nearly 90% of DPA micelles emission was quenched by small amount of EY, showing the efficient FRET. It was estimated that 48 donors can funnel their energy to a single acceptor with >50% efficiency, suggesting antenna effect of 7.6 (estimation according to Equation 10).

Our group developed fluorescent micelles that are about the size of proteins. These nanoparticles were synthesized by forming a covalent corona of PEGylated cyanine bis‐azides (Cy3L and Cy5L) around micelles made from amphiphilic calixarene with alkyne groups (Figure 10c).^[^ ^229^ ^]^ The resulting nanoparticles, which measured around 7 nanometers in diameter according to AFM (Figure 10d), were approximately twice as bright as quantum dots 585 with a QY of up to 15%. The micelles cross‐linked by Cu‐catalyzed click reaction with Cy3L (donor) and Cy5L (acceptor) showed dual emission dependent on the donor/acceptor ratio, while the control without Cu catalyst showed no FRET signal (Figure 10e). At donor/acceptor ratio of 50/1, the cross‐linked micelles displayed 69% FRET efficiency,^[^ ^229^ ^]^ suggesting (according to Equation 10) antenna effect of 21. FRET studies indicate that these nanoparticles were very stable in both aqueous and organic environments, as well as within living cells, where they provided a good imaging contrast.^[^ ^229^ ^]^

**Figure 11:**
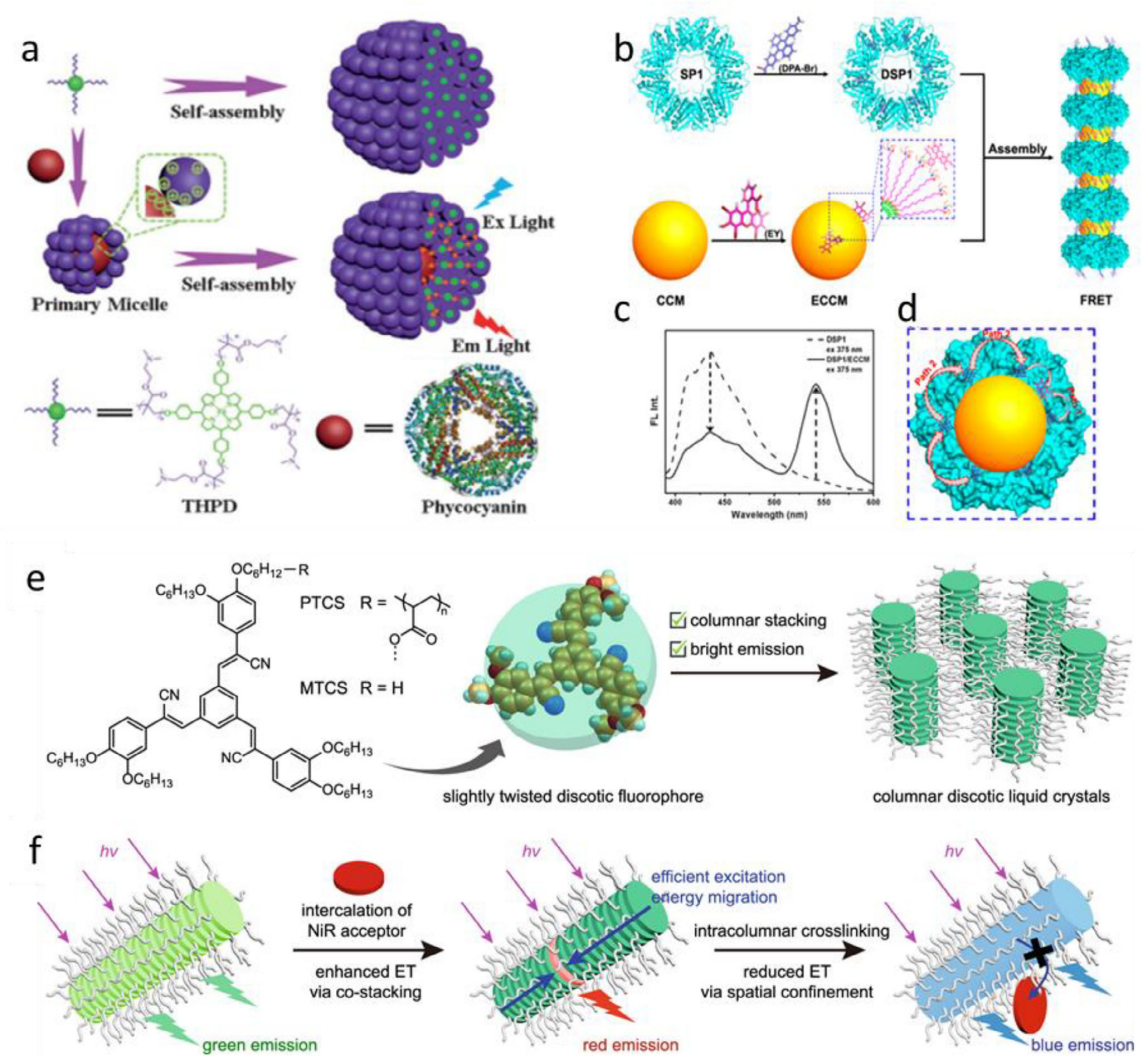
a) Self‐assembly of the pomegranate‐like porphyrin‐phycocyanin light‐harvesting nanosystem (Ex Light = exciting light; Em Light = emitted light). Reproduced with permission.^[^ ^230^ ^]^ Copyright 2016, John Wiley and Sons. b) Design of light‐harvesting nanowires. Assembly model of DSP1/ECCMs nanowire with DSP1 (cyans) and ECCMs (yellow). Donor, DPA (purple), is located on the top and bottom surfaces of SP1. Acceptor, EY (rose), is modified on the surface of CCMs by electrostatic interactions. c) Fluorescence spectra of DSP1/CCMs and DSP1/ECCMs upon excitation at 375 nm. d) For systems with large numbers of donors, energy can be transferred to acceptor chromophores via direct donor‐to‐energy transfer (Path 1) or multistep donor‐to‐donor transfers (Path 2). Reproduced with permission.^[^ ^232^ ^]^ Copyright 2016, American Chemical Society. e) Molecular structures of PTCS and MTCS, which are utilized to construct supramolecular columns. The slightly twisted discotic geometry of the TCS fluorophore facilitates self‐assembly into columnar LC phases and preserves bright emission. f) Modular columnar assembly enables efficient and controllable ET behaviors. The neat PTCS supramolecular columns exhibit bright green light emission. Co‐stacked PTCS‐NiR supramolecular columns display red emission with enhanced ET character due to the efficient excitation energy migration along the columns. Intracolumnar crosslinking of TCS fluorophores leads to blue fluorescence by reducing ET via spatial confinement. Reproduced with permission.^[^ ^236^ ^]^ Copyright 2024, Springer Nature.

Zhou et al reported an LHS based on protein framed multiporphyrin micelles. The core of the micelle was formed by phycocyanin acceptor, while the shell was assembled from a four‐armed porphyrin molecule with Zn(II) core (THPD) (**Figure** **11**a) due to electrostatic interactions. The obtained micelles of 76 nm diameter showed FRET efficiency of 80.1%. It was estimated that 179 donors contributed their collective energy to a single acceptor in this nanosystem confirming an efficient LHS (Figure 11a).^[^ ^230^ ^]^ Assuming the extinction coefficient of photophyrin and phycocyanin^[^ ^231^ ^]^ of 4 × 10^5^ and 1.54 × 10^6^ M^−1 ^cm^−1^, the estimated antenna effect in this system should reach 37.

Liu et al. fabricated 10 nm core‐cross‐linked micelles with cationic surfactant, (11‐acrylatylundecyl)triethylammonium bromide.^[^ ^232^ ^]^ They were self‐assembled with cricoid stable protein one (SP1) via electrostatic interactions into nanowires (Figure 11b). These micelles can interact with two central cavities SP1 at the opposite sides of the protein toroid forming a sandwich like structure, which propagates in 1D in form of a nanowire. The SP1 was labelled with 9‐[4‐(bromomethyl)‐ phenyl]‐10‐(4‐methylphenyl) anthracene (DPA‐Br), which acted as donor, whereas the micelles were labelled with EY dye. The absorbed energy by DPA is then transferred to EY by FRET, which was evidenced by the increase in the acceptor emission peak and donor emission quenching (Figure 11c,d). The donor dyes underwent homo‐FRET 59% efficiency which was accompanied by hetero‐FRET with 52% efficiency.^[^ ^232^ ^]^ These artificial LH nanowires mimic natural LH‐2 complex.

More recently, an artificial LHS was built though micellar assembly of a hexameric hemoprotein modified with poly(N‑isopropylacrylamide).^[^ ^233^ ^]^ The latter ensured assembly into ≈43‐nm micelles, which could be further cross‐linked into stable LH nanoparticles containing large number of copies of photophytin dyes. Remarkably, nanoparticles containing Zn‐photophytin showed fast EET within the dyes on the time scale of several tens of picoseconds. These results indicated strong potential of these materials for LH applications, although the FRET to an acceptor dye has not been studied here.

The amphiphilic dye may lead to high dimension LH nanomaterials, for example 2D assemblies. Thus, Rybtchinski and co‐workers employed an amphiphilic perylene diimide to assemble crystalline nanosheets.^[^ ^234^ ^]^ They presented broad absorption, important for panchromatic light energy collection and excitation energy transfer on the ps time scale, with exciton diffusion length reaching 120 nm.

In the further effort to mimic natural LHS in cyanobacteria, a sequential energy transfer system was constructed via supramolecular copolymerization of σ‐platinated (hetero)acenes: anthracene, tetracene, and naphtho[2,3‐c][1,2,5]selenadiazole, emitting in green red and near infrared regions.^[^ ^235^ ^]^ The resulting supramolecular copolymers in form of helixes displayed long exciton migration length of >200 donor units and high EET rates, which resulted in overall sequential energy transfer efficiency of 87.4% for the ternary copolymers. Very recently, Tian and co‐workers developed slightly twisted non‐planar discotic tricyanotristyrylbenzene (TCS), which assembled into supramolecular polymeric columns, which bio‐mimicked natural LHS (Figure 11e,f).^[^ ^236^ ^]^ When acceptor dye Nile Red was intercalated in these columns, an efficient FRET (≈40%) was achieved at donor acceptor ratio of 1000:1. Remarkably, FRET was observed even at donor: acceptor ratio of 20 000:1, which resulted in the an antenna effect exceeding 100.^[^ ^236^ ^]^

#### 4.6.3 Dye‐Doped Silica Nanoparticles

Dye‐doped silica nanoparticles attracted significant interest due to their tunable size, low toxicity and remarkable optical properties,^[^ ^237^, ^238^ ^]^ which nurtured various biological applications.^[^ ^239^, ^240^, ^241^ ^]^ In dye‐loaded silica nanomaterials (DLSNs) from mesoporous silica, the pore size can be varied in broad range from 2 to 50 nm,^[^ ^241^, ^242^ ^]^ which is perfect for dye encapsulation, whereas the surface chemistry is well studied and ease of surface modification.^[^ ^239^ ^]^

**Figure 12:**
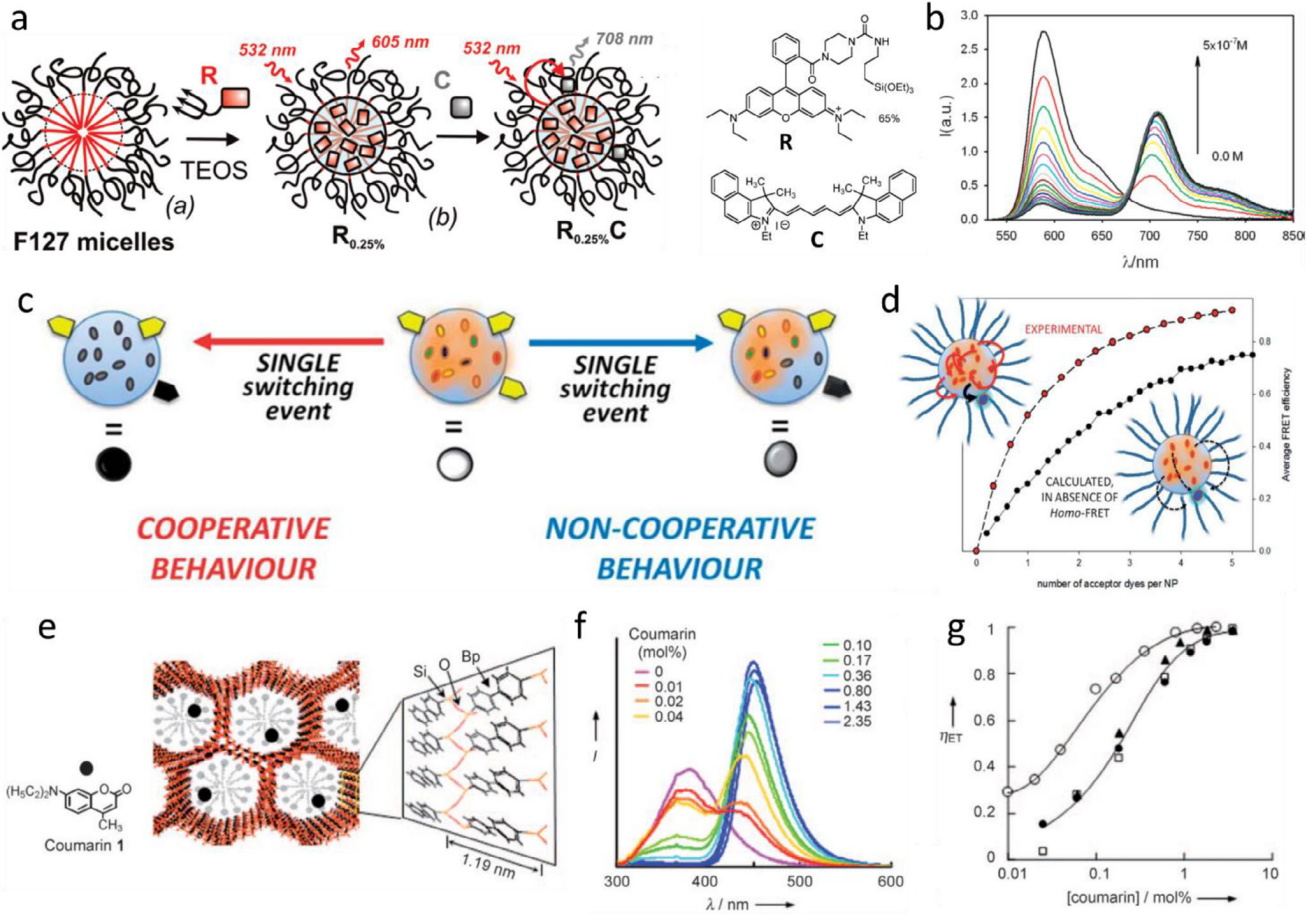
a) Schematic representation of the synthesis of NPs R0.25% using F127 micelles as template, and added acceptor molecules of cyanine (C) are hosted by the NPs that quench the donor rhodamine (R) fluorescence by FRET. b) Fluorescence spectra of a 1 × 10^−7^ M water solution of NPs R0.25% during the titration with C upon excitation at 532 nm. Each variation corresponds to the addition of 3.3 × 10^−8^ M of C. Reproduced with permission.^[^ ^243^ ^]^ Copyright 2010, American Chemical Society. c) Schematic illustration of cooperative and non‐cooperative effects in dye‐doped nanostructures. Reproduced with permission.^[^ ^245^ ^]^ Copyright 2014, the Royal Society. d) Experimental FRET efficiency (red dots) versus simulated average FRET efficiency (black dots). No homo‐FRET between donors is considered in the simulations. Each point represents the average FRET efficiency for a set of 1000 NPs containing 10 donor dyes on average embedded in a 5.5 nm radius core, and an increasing number of acceptor dyes hosted in a very thin shell (0.2 nm). Reproduced with permission.^[^ ^245^ ^]^ Copyright 2014, the Royal Society. e) Energy‐transfer properties of coumarin 1 dye‐doped biphenyl‐PMO. Schematic representation of coumarin 1/biphenyl‐PMO. Bp = biphenyl. f) Fluorescence spectra for coumarin 1/biphenyl‐PMO powders with 0–2.35 mol% coumarin 1 (excitation wavelength, 270 nm; normalized by absorption rate). g) Energy‐transfer efficiency of biphenyl‐PMO powder, and biphenyl‐PMO films prepared using three different template surfactants: P123 (wormhole; □), C18TMACl (lamellar; ∆), and Brij76 (lamellar; ●). Reproduced with permission.^[^ ^247^ ^]^ Copyright 2009, John Wiley and Sons.

Pluronic silica (PluS) NPs developed by Prodi et al. is a nice example of DLSN with high cooperativity (**Figure** **12**a,b).^[^ ^240^ ^]^ These NPs were obtained by direct micelle‐assisted method, using Pluronic F127 (polyethyleneglycol–polypropylene oxide–polyethyleneglycol (PEG–PPO–PEG) amphiphilic structure) as a surfactant. These NPs displayed a 10 nm diameter of their silica core with overall hydrodynamic diameter of 25 nm. The confined rhodamine B dyes inside PluS NPs exhibited high cooperativity, because homo‐FRET distributed the excitation energy over at least 4 donor dyes. it was found that 10 rhodamine B donor dyes can be quenched ≈90% in presence of a single cyanine 5 dye (acceptor), which suggests efficient energy transfer,^[^ ^243^ ^]^ with estimated antenna effect (by Equation 10) of ≈4.5. The same group then used this efficient FRET within silica NPs, to decrease self‐quenching within donor dyes and thus enhance particle brightness.^[^ ^244^ ^]^ In a review by the same group made, a theoretical consideration of silica NPs with low and high cooperativity were made.^[^ ^245^ ^]^ For non‐cooperative systems FRET takes place independently for each donor dye to an acceptor. However, in this case the estimated FRET efficiency is significantly lower compared to the experiment (Figure 12c,d). In the cooperative behavior the donor dyes communicate due to efferent EET, which favors the efficient FRET from a large ensemble of the encapsulated dyes to few acceptors.

Cucinotta et al. fabricated an LH antenna using mesoporous organosilica material. BODIPY dyes were doped by self‐assembly with surfactant, forming micellar structures and thereafter silica NPs.^[^ ^246^ ^]^ Two derivatives of BODIPY were tested: one modified with PEG and another one bearing octyl chain. The former shows a distinct green fluorescence which gets slightly red shifted and quenched with increasing dye loading in the system. In contrast, the second dye resulted in NPs with polychromatic emission dependent on the loading. Here, the monomeric high energy species transferred energy efficiency to the aggregated low energy species, leading to red shifted emission band at higher dye loading. The efficiency of the LHS was confirmed by the fast energy migration kinetics with the time scale of 20 and 80 ps.^[^ ^246^ ^]^

An efficient periodic mesoporous organosilica‐based LHS can be realized by hosting two dyes into different compartments (walls and meso‐channels), as shown by Inagaki and co‐workers.^[^ ^247^ ^]^ Biphenyl dye (donor) was hosted in the organosilica walls, while the coumarin dye (acceptor) was encapsulated inside the meso‐channel. Then the FRET efficiency was measured in both films and in amorphous form. With increase in the amount of coumarin acceptor, its emission intensity increased, while donor intensity decreased. The total QY increased twice with FRET efficiency reaching ≈100%. The same group also reported acridone‐bridged periodic mesoporous organosilica nanomaterials, which showed efficient light‐harvesting and energy transfer to (4‐(dicyanomethylene)‐2‐methyl‐6‐(p‐dimethylaminostyryl)‐4H‐pyran) (DCM) dye encapsulated at 1 mol%. Latter resulted in six‐fold enhancement in QY (36%) with emission at 600 nm. Other examples of this approach were summarized in a review by Rao et al.^[^ ^248^ ^]^ Overall, dye‐doped silica nanomaterials are excellent materials for LHS preparation, which were already proposed for amplified sensing applications.^[^ ^245^ ^]^

#### 4.6.4 Dye‐Loaded Polymeric Nanoparticles

Dye‐loaded polymeric NPs are particularly suitable for efficient light harvesting due to their capacity to encapsulate large amounts of dyes and tunability of their size and surface properties.^[^ ^23^, ^29^, ^249^ ^]^ Moreover, to prevent ACQ at high loading a number of effective strategies particularly suitable for polymeric NPs were developed, such as AIE, bulky side groups or a bulky hydrophobic counterion.^[^ ^23^ ^]^ Earlier, Law and co‐workers introduced polymeric NPs loaded with cyanine dyes in order to achieve efficient cascade FRET. DiD, DiO and DiI dyes were encapsulated into poly(D,L‐lactic‐co‐glycolic acid) (PLGA) nanoparticles with polyethylene glycol (PEG) shell.^[^ ^250^ ^]^ They showed that FRET can be efficient between donor and acceptor at 0.5‐2 wt% loading and ratios donor to acceptor 1:1 to 1:20. Remarkably, a cascade FRET for up to four fluorophores were achieved from green DiO to DiI followed by DiD and then DiR, leading to impressive Stokes shift in the emission, useful for bioimaging. However, in these studies, it was shown that the self‐quenching become important from 0.5 wt% of the donor dye,^[^ ^50^ ^]^ which is detrimental for construction of efficient LHS.

**Figure 13:**
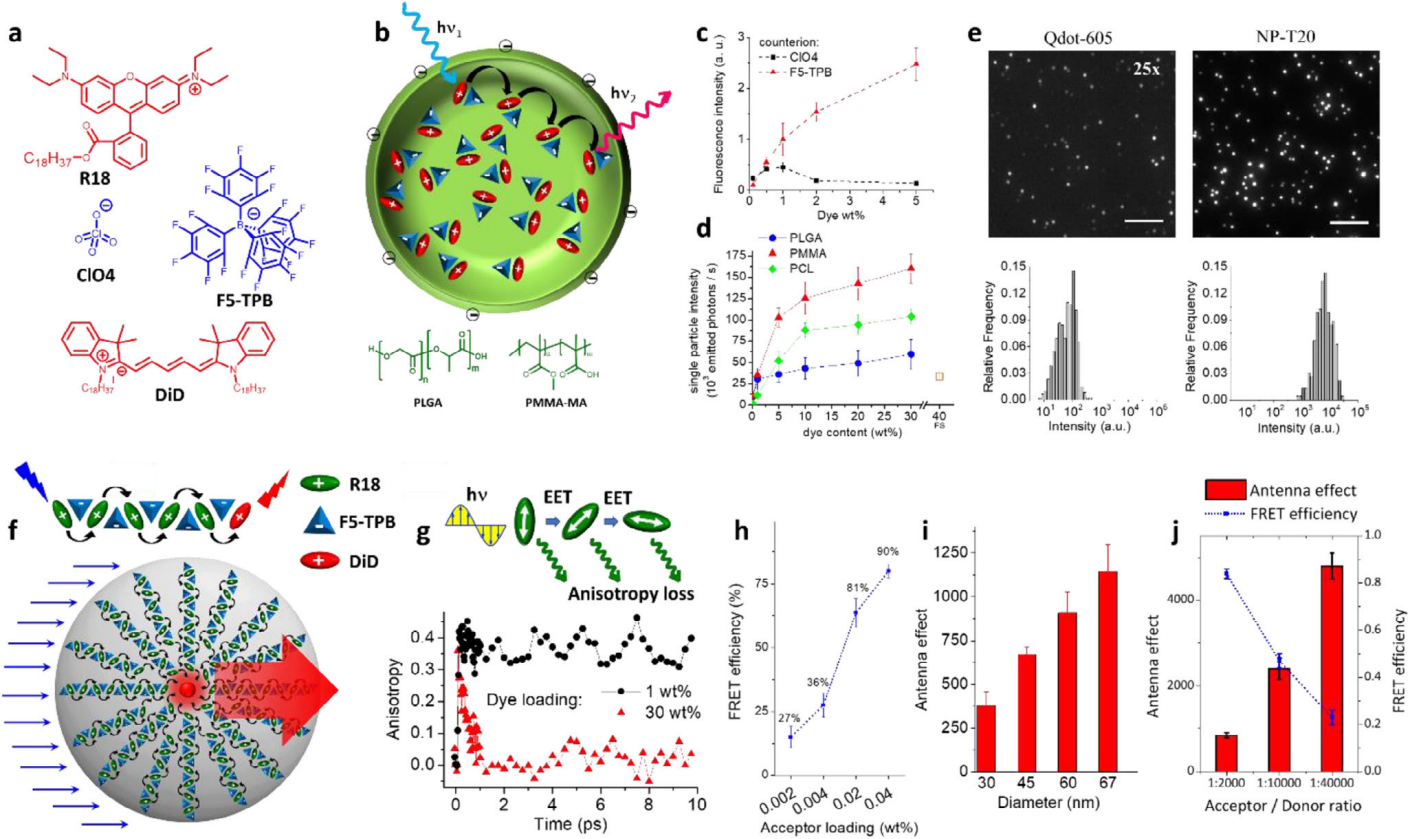
Design and properties of dye‐loaded polymer NPs using bulky counterions. a) Chemical structures of rhodamine B octadecyl ester (R18) and its different counterions: perchlorate (ClO4), and tetrakis(pentafluorophenyl)borate (F5‐TPB). b) Schematic representation of PLGA NPs loaded with these dye salts. c) Fluorescence intensity of PLGA NPs versus dye content of R18 with different counterions. Reproduced with permission.^[^ ^36^ ^]^ Copyright 2014, Springer Nature. d) Particle brightness versus dye loading for NPs build of different polymers. Reproduced with permission.^[^ ^251^ ^]^ Copyright 2017, American Chemical Society. e) Wide‐field fluorescence microscopy of the immobilized NPs: quantum dots (QDot‐605) excited at 470 nm and 40‐nm PMMA‐based NPs loaded with 23 wt% of R18/F5‐TPB coated with T20 oligonucleotides (NP‐T20). To obtain comparable signals excitation power density for QDot‐605 was 25‐fold higher than that for NP‐T20 and nanoprobe: 14 versus 0.56 W cm−2, respectively. (c) Histogram of single‐particle intensity distribution for these three types of NPs. At least 1000 NPs were analyzed in each case. Reproduced with permission.^[^ ^71^ ^]^ Copyright 2018, American Chemical Society. f) Schematic representation of giant‐light harvesting nanoantenna based on PMMA‐MA NPs encapsulating containing donors (R18/F5‐TPB) and the acceptor (DiD/F5‐TPB). g) Time‐resolved fluorescence anisotropy of the PMMA‐MA NPs of 44 nm size (TEM) at different donor dye loading. h) FRET efficiency 44‐nm PMMA‐MA NPs with 30 wt% R18/F5‐TPB (versus polymer) versus acceptor (DiD/F5‐TPB) dye loading. i) Antenna effect for PMMA‐MA NPs of different size (by TEM) loaded with 30 wt% R18/F5‐TPB with donor/acceptor ratio 10000:1. Reproduced with permission.^[^ ^98^ ^]^ Copyright 2017, Springer Nature. j) Antenna effect and FRET efficiency of 70‐nm PMMA‐MA NPs with different acceptor (DiD/F5‐TPB) to donor (R18/F5‐TPB) ratios co‐encapsulated with BHS. Reproduced with permission.^[^ ^258^ ^]^ Copyright 2024, John Wiley and Sons.

In 2014, our group pioneered a solution to the ACQ problem in dye‐loaded polymeric NPs using bulky hydrophobic counterions.^[^ ^36^ ^]^ Here, cationic dye rhodamine B dye with long alkyl chain (R18) was paired with bulky hydrophobic tetraphenylborate counterions and then encapsulated inside 40‐nm PLGA NPs (**Figure** **13**a,b). The latter were prepared by nanoprecipitation, where a single charge in PLGA chain‐controlled formation of small NPs. Among studied TPB, the one with highest level of fluorination (F5‐TPB) showed remarkable capacity to minimize ACQ: the fluorescence brightness of NPs increased with R18/F5‐TPB ion par loading up 5wt% (QY = 21%), whereas the same dye R18 with perchlorate counterion showed strong ACQ above 1 wt% loading (Figure 13c).^[^ ^36^ ^]^ These NPs were 6‐fold brighter than QDs‐585 at 532 nm excitation in single‐particle microscopy. Moreover, they showed complete ON/OFF switching of particle fluorescence. According to lifetime anisotropy, the latter was caused by fast dye‐dye communication, i.e., homo transfer of energy (on the timescale beyond the resolution of instrument, <20 ps), suggesting high cooperativity of dyes inside NPs.^[^ ^36^ ^]^ Then, the use of more hydrophobic polymer, poly(methyl methacrylate)‐co‐methacrylic acid (PMMA‐MA) allowed further reducing ACQ and thus reaching QY about ≈50% even at 23 wt% of R18/F5‐TPB dye loading (30 wt% versus polymer, Figure 13d) in 34‐nm NPs.^[^ ^251^ ^]^ This improvement was attributed to more homogeneous distribution of hydrophobic dyes in more hydrophobic polymer. Even though PMMA‐MA NPs showed much smaller ON/OFF switching, at the highest studied dye loading emission intermittency was observed, which confirmed the fast energy migration in these NPs.^[^ ^251^ ^]^ Singe‐particle microscopy studies also showed that the obtained PMMA‐MA NPs were nearly 100‐fold brighter than corresponding QD‐605 excited at 470 nm (Figure 13e).^[^ ^71^ ^]^ Ultimately, we further decreased the hydrophobicity of the polymer by replacing PMMA‐MA with poly(ethyl methacrylate)‐co‐methacrylic acid (PEMA‐MA). It enabled preparation of 20 nm NPs with 33 wt% of R18/F5‐TPB dye with respect to the total mass of the NPs (50 wt% versus polymer mass), characterized by higher QY (52%) and lower blinking than PMMA‐MA based NPs.^[^ ^252^ ^]^

It is important to stress that at high dye loading (e.g., 23 wt% dye loading versus total NP mass) the effective dye‐dye distance should be reduced to <2 nm. This spatial proximity of dyes should facilitate the EET. According to the ultrafast pump‐probe measurements, the donor‐donor energy transfer (EET) in these NPs took place at the time scale of 30 fs (Figure 13f,g).^[^ ^98^ ^]^ This exceptionally fast EET together with high QY should ensure that within the lifetime the excitation energy migrates within thousands of dyes, i.e., all encapsulated dyes, which is prerequisite of a powerful LHS. Recent studies suggested that this collective behaviour, characterized by incoherent exciton hopping, resulted in a high exciton diffusion constant (> 0.003 cm^2^/s) and a long exciton diffusion length (≈70 nm) corresponding to the particle size.^[^ ^253^ ^]^ These features enable the delocalization of excitonic energy across the donor network within the nanoparticle. To explore LH properties of these NPs, we prepared PMMA‐MA NPs containing large amount of donors and few acceptors at ratio 1000:1 and 10000:1.^[^ ^98^ ^]^ Even at the donor‐to‐acceptor ratio 10000:1 (acceptor loading was 0.002 wt%), which corresponded statistically to a single acceptor per particle, efficient FRET (27%) was observed (Figure 13h). For the constant donor:acceptor ratio 10000:1 the antenna effect increased with the size of PMMA‐MA NPs, reaching antenna effect of 1150 for 67 nm NPs (Figure 13i),^[^ ^98^ ^]^ which was by far the largest value reported to date. Due to this giant signal amplification by LHS, one acceptor dye showed comparable emission intensity as the whole NP with ≈10 000 donors. After the single‐step bleaching of the acceptor the donor NPs revived back its emission in the one step process. Moreover, this giant LH nanoantenna enabled unprecedented single molecule (acceptor) detection under excitation equivalent to ambient sunlight. Effective LH properties of dye‐loaded polymeric NPs were directly applied for controlled particle switching, where small quantity of encapsulated photo‐switchable dyes turned ON/OFF large number of donor dyes inside particle under light stimulus.^[^ ^254^ ^]^ Remarkably, the developed giant light‐harvesting nanoantenna became a powerful platform for development of nanoprobes for amplified biosensing,^[^ ^71^, ^252^, ^255^, ^256^, ^257^ ^]^ which will be explained below.

Recently, our group reported that adding blank hydrophobic salts (BHS) composed of the same bulky counterion (F5‐TPB) paired with an optically inactive cation can further reduce ACQ. BHS doubled the fluorescence QYs and lifetimes of the NPs and reduced fluorescence blinking. As a result, efficient FRET was achieved, allowing energy transfer from 40 000 donor R18/F5‐TPB dyes (loaded at 50 wt% with respect to total NP mass) to a single acceptor within a 70 nm particle (Figure 13j, it is worth to note that the number of acceptors per NP is a statistical value, which follows Poisson distribution). The obtained material displayed a record‐breaking antenna effect of 4800 (Figure 13j).^[^ ^258^ ^]^ Utilizing this enhanced nanoantenna, single‐molecule detection of the FRET acceptor was successfully demonstrated at low excitation power using an RGB camera from a smartphone.^[^ ^256^, ^258^ ^]^

**Figure 14:**
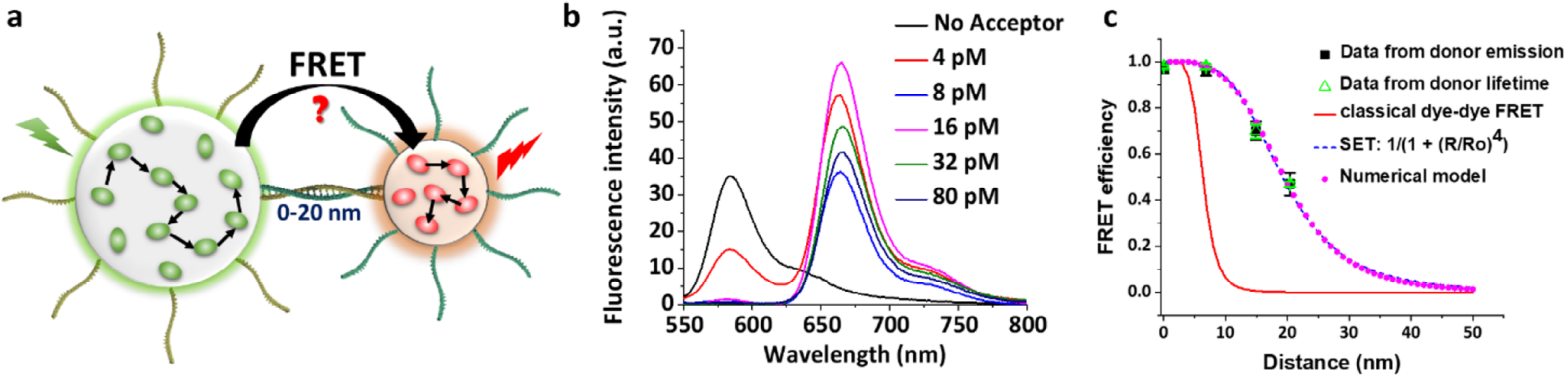
Energy transfer between two dye‐loaded polymeric NPs (a) Schematic presentation of two DNA‐modified NPs loaded with donor (R18/F5‐TPB) and acceptor (Atto647‐C18/F5‐TPB) dyes. The distance between donor and acceptor NPs are controlled by the length of DNA duplexes. b) Fluorescence spectra of donor DNA‐modified NPs (40 nm NPs containing 50 wt% of R18/F5‐TPB dye with respect to the polymer) at 8 pM concentration incubated with increasing concentrations of acceptor NPs (25 nm NPs containing 30 wt% ofAtto647‐C18/F5‐TPB dye with respect to the polymer). c) FRET efficiencies between these NPs measured using both steady‐state (black squares) and time‐resolved (green triangles) spectroscopy as a function of their surface‐to‐surface distance. Reproduced with permission.^[^ ^262^ ^]^ Copyright 2023, John Wiley and Sons.

One of the very long‐standing questions is the distance dependence of FRET between two nanoparticles. The Forster formalism, where FRET efficiency decays with a power six law, considers donor and acceptor as two‐point dipoles.^[^ ^22^, ^32^ ^]^ However, these may not apply in case of nanomaterials that cannot be considered as point dipoles. Previously, the deviation of standard FRET model has been described for gold plasmonic systems, where FRET efficiency decayed with distance to the fourth power.^[^ ^259^ ^]^ For non‐plasmonic systems, the distance dependent FRET is supposed to be controlled by the geometries of the systems.^[^ ^260^, ^261^ ^]^ Medintz and co‐workers recently showed a power four distance dependence with an artificial LHS made by DNA bricks.^[^ ^210^ ^]^ It was shown that in case of energy transfer between surfaces the power six law may change to power four law. Moreover, this studies showed that increasing number of acceptors can enhance the FRET efficiency of the system. Recently, we studied FRET between two dye‐loaded NPs (**Figure** **14**a):^[^ ^262^ ^]^ one loaded with donor dyes (R18/F5‐TPB) and another loaded with acceptor dyes (hydrophobic ATTO647N derivative with F5‐TPB). They were connected with DNA duplexes, in order to tune the NP‐NP distance. Remarkably, they show nearly 100% FRET efficiency for 20‐nt duplex linker, corresponding to 6.8 nm distance (Figure 14b). Given that this distance is close to the Forster radius for this dye pair (6.35 nm), the obtained nearly quantitative FRET efficiency was outstanding. Further increase in duplex length up to 60 nucleotides, corresponding to 20.4 nm distance, led to FRET efficiency of 45%, which showed that this two‐particle system undergoes unexpectedly long‐distance FRET. It was found that the FRET efficiency follows power‐four dependence on the NP‐NP surface to surface distance (Figure 14c), in contrast to power‐six Forster law. This was then theoretically explained by point donor to 2D half sphere acceptor model. In the half sphere, it is considered that acceptor dyes are homogeneously distributed at the surface of the acceptor NPs. But to match the experimental observations, 10000 acceptor dyes were required but our acceptor NP of 26 nm contained only 800 dyes. Here, two additional points explain this remarkable behavior: (i) the donor is not a point donor but a large dye ensemble strongly coupled by ultra‐fast EET and confined in sphere of 40 nm diameter. In this system, after electronic excitation EET can bring energy to practically any donor dye located at the surface close to acceptor NPs, favoring efficient FRET. On the other hand, in the acceptor NPs also, due to fast EET, the transferred excitation energy gets rapidly redistributed within 800 dyes of the acceptor NP. Overall, combining the two LHS systems breaks the Förster law and allows energy transfer over remarkably long distance, namely up to 50 nm distance between centers of two NPs (20 nm surface to surface distance) with 45% efficiency (Figure 14c).

## 5 Light‐Harvesting Dye‐Based Materials for Amplified Biosensing

### 5.1 Signal Amplification in Biosensing

The emerging field of personalized medicine requires substantial improvement and development of advanced diagnostics tools for point‐of‐care surveillance. To provide a fast response to the outbreak of diseases, novel diagnostic tools should correspond to the following criteria: affordable, sensitive, specific, user‐friendly, equipment‐free, and delivered to those who needed.^[^ ^263^, ^264^ ^]^ In this respect, nanotechnology‐enabled (bio)sensors, which are nanoscale devices that couple recognition of (bio)molecule with a detectable output signal, are of particular importance.^[^ ^265^, ^266^, ^267^ ^]^ The eventual goal in the development of nano‐biosensors is to achieve an ultrasensitive response to the target in the simplest nanodevice with minimal sample treatment, with an ultimate goal – single‐molecule detection. However, a very low concentration of molecules is unable to generate a sufficient signal for detection with conventional optical methods. One of the obvious solutions is to use amplification techniques, where a small input signal is transformed into a large output signal with the help of an amplifier.^[^ ^6^, ^268^ ^]^ In optical biosensors, all amplification strategies can be divided into two principal categories: amplification achieved via: i) molecular or chemical multiplication and ii) signal amplification using physical principles of amplification. The molecular multiplication strategy leads to the increasing number of molecules created after a recognition event. It can be a replication of target molecules before detection or replication of labels, which leads to an increase in the number of signal‐producing analyte recognition events. The classical example of molecular amplification system for nucleic acids is polymerase chain reaction (PCR). It exploits replication of target strands of DNA with the help of enzymes (DNA polymerases) prior to detection and a thermocycler as each step of PCR requires different temperatures. After 30 cycles of amplification billions of DNA copies are produced, which followed by post‐PCR steps that include analysis of amplified products.^[^ ^269^ ^]^ However, this method requires dedicated instrumentation, experienced staff and expensive reagents and, thus, it is difficult to translate it to the point‐of‐care testing. Therefore, isothermal amplification methods appeared as an alternative, where one temperature is used for the entire amplification process. Particularly popular are loop‐mediated isothermal amplification (LAMP),^[^ ^270^, ^271^ ^]^ rolling cycle amplification (RCA),^[^ ^272^, ^273^ ^]^ etc. However, similarly to PCR those are indirect methods, which require heating, specific enzymes as well as reverse transcription in case of RNA detection.

Alternative approach is to use optical amplification that enhances signal for detection by physical mechanisms. In optical amplification, signal is amplified by an optical antenna, which can convert molecular recognition event into strong optical response of the biosensor device.^[^ ^6^, ^103^, ^274^ ^]^ Two main mechanisms of optical amplification should be mentioned: i) plasmonic materials‐based signal enhancement; ii) LHS‐based signal amplification.

Plasmonic nanomaterials (gold or silver nanoparticles) are known to greatly enhance the fluorescence signal of organic dyes with optimal position and orientation.^[^ ^275^ ^]^ This enhancement has several applications in various fields with biosensing with signal amplification.^[^ ^103^, ^104^, ^105^, ^276^, ^277^, ^278^ ^]^

LHS‐based signal amplification uses energy transfer from nanoantenna to the sensing unit. In this case, sensing event either modulates FRET efficiency or switches emission of the acceptor, both resulting in the robust ratiometric response to the analyte. As FRET from the antenna couples a large number of donors to a few acceptors, a single molecular recognition event can result in a response of the large ensemble of donor dyes or change in the acceptor emission amplified the nanoantenna. Therefore, these systems produce signal amplification, which can improve sensitivity of the nanoprobes compared to corresponding molecular probes. The primary examples of nanoprobes that used this signal amplification principle wer conjugated polymer NPs (CPNs), which have already been extensively reviewed.^[^ ^5^, ^6^, ^7^ ^]^ Therefore, in this review we will only concentrate on dye‐based systems and we will discuss about the sensing applications for detection of small and large (bio)molecules.

In a nanoprobe, LHS is functionalized with a analyte‐sensing unit, which is generally used as a FRET acceptor. This configuration is the most optimal, because LHS can then amplify the emission of the sensing unit. As biological applications deal with aqueous media, it is of outmost importance that the nanoprobe is stable in aqueous buffers, which implies chemical and colloidal stability. Finally, when applied to cells, the nanoprobe should be biocompatible and non‐toxic as well as present minimal non‐specific interactions with off‐target biomolecules.

### 5.2 Detection of Small Molecules and Ions

**Figure 15:**
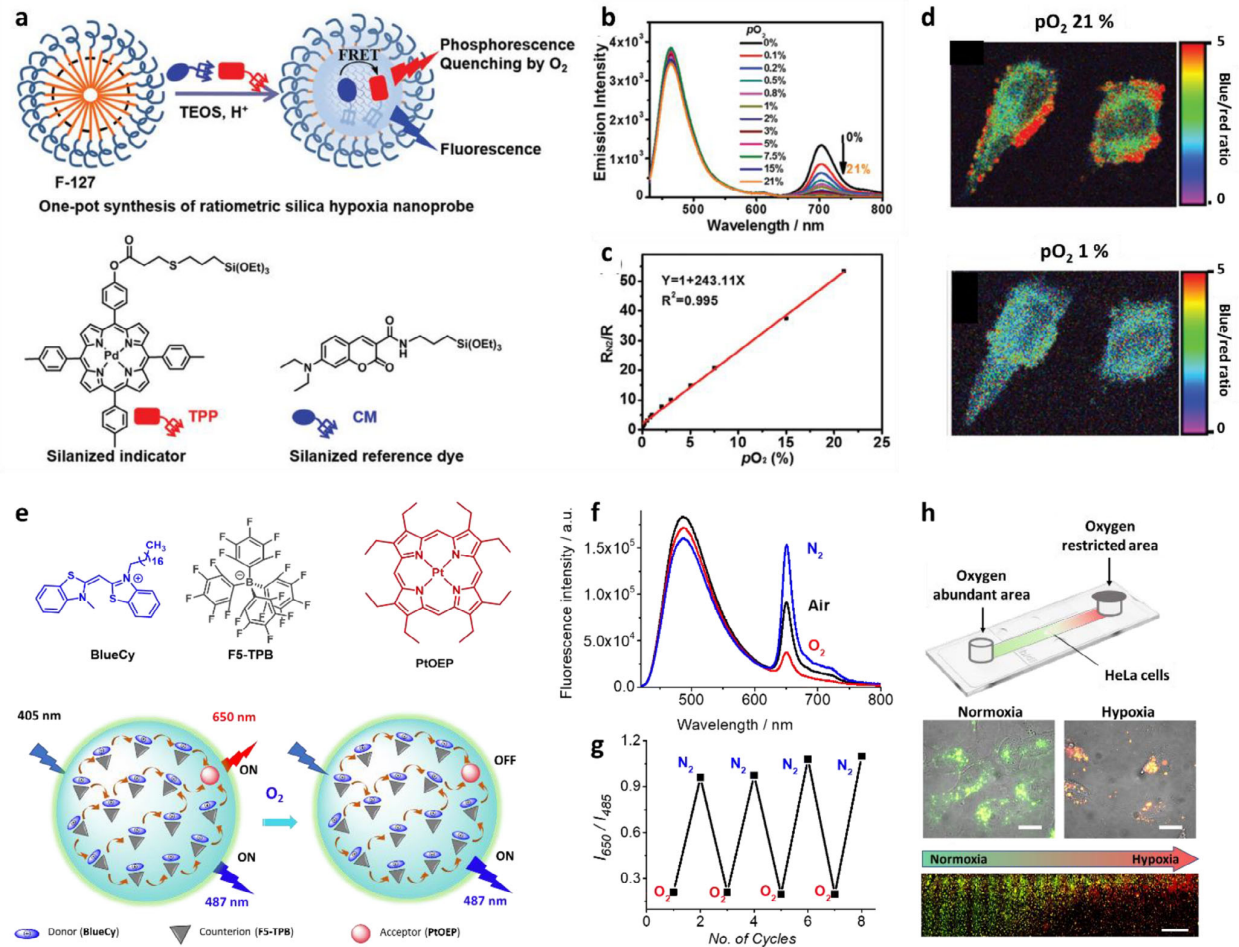
a) Schematic illustration of the one‐pot preparation of ratiometric hypoxia nanoprobes based on covalent‐bonded silica nanoparticles (NPs); the chemical structures of the oxygen‐sensitive indicator (TPP) and oxygen‐insensitive reference dyes (CM). b) Ratiometric emission spectral response of the silica nanoprobe in aqueous solution at different oxygen levels (0%, 0.1%, 0.2%, 0.5%, 0.8%, 1%, 2%, 3%, 5%, 7.5%, 15%, 21%), λ_ex_ = 410 nm. c) Linear Stern–Volmer plot of R_N2_ /R as a function of oxygen partial pressure. d) Confocal luminescence ratiometric luminescence images (λ_ex_ = 405 nm) of living HeLa cells incubated with the silica nanoprobe (1.4 nM) at 21% (top) and 1% (bottom) oxygen partial pressure. The ratio of emission intensity at 430–470 nm to that at 660–740 nm (I_blue_/I_red_) was chosen as the detected signals. Reproduced with permission.^[^ ^283^ ^]^ Copyright 2021, the Royal Society. e) Concept of light‐harvesting nanoparticle encapsulating BlueCy‐TPB/PtOEP for ratiometric detection of oxygen. Chemical structures of the cyanine donor dye (BlueCy) with its counterion F5‐TPB and of the porphyrin acceptor dye (PtOEP) and schematic representation of FRET‐based ratiometric oxygen nanoprobe. Oxygen sensing experiments using BlueCy‐TPB/PtOEP NPs in phosphate buffer solution at pH 7.4. f) Ratiometric response in emission spectra of BlueCy‐TPB/PtOEP NPs toward the change in oxygen concentration in the solution. Oxygen concentration was varied by purging oxygen and nitrogen gases. g) Ratio of emission intensities between acceptor and donor for alternative purging of oxygen and nitrogen cycles, to confirm the stability and reversibility of the BlueCy‐TPB/PtOEP NPs as the oxygen probe. In vitro imaging of oxygen gradient in a microfluidic plate using BlueCy‐TPB/PtOEP NPs with wide‐field fluorescence microscopy. h) Schematic representation of stable oxygen gradient generation in the microfluidic slide and fluorescence microscopic images of HeLa cells after incubation for 3 h with BlueCy‐TPB/PtOEP NPs. The merged images obtained by merge of green (donor) and red (acceptor) and transmission (grey) channels in the oxygen‐abundant area (normoxia) and in oxygen‐restricted area (hypoxia) of the µ‐slide. Scale bar: 10 µm. Lowest panel: large‐scale image from one end to other end of the µ‐slide using 10X objective, which clearly depicts the oxygen gradient. Scale bar: 500 µm. Reproduced with permission.^[^ ^257^ ^]^ Copyright 2020, John Wiley and Sons.

The ability to sense small molecules with high selectivity and sensitivity is very important for the biology.^[^ ^247^ ^]^ There is a number of light‐harvesting sensors for detection of small molecules, such as oxygen,^[^ ^279^ ^]^ reactive oxygen species (ROS),^[^ ^248^, ^249^, ^250^ ^]^ glucose,^[^ ^251^ ^]^ neurotransmitters,^[^ ^252^, ^253^ ^]^ ions,^[^ ^254^ ^]^ etc. Sensing oxygen is of particular importance in biomedical context, as lack of oxygen (hypoxia) frequently associated with a number of pathologies related to stroke, cardiovascular diseases and cancer. Therefore, significant efforts were done to develop optical probes for oxygen.^[^ ^279^ ^]^ Here, light harvesting can serve to enhance the signal of oxygen‐sensitive units, such as phosphorescent porphyrin‐metal complexes. An early example is dendritic system, where porphyrin molecule, having poor two‐photon absorption was functionalized with multiple coumarin groups as energy donors, featuring strong two‐photon absorption.^[^ ^280^ ^]^ Thanks to light harvesting by the donors and efficient FRET to the porphyrin acceptor, two‐photon imaging of oxygen distribution was performed in cerebral vasculature and tissue. So far, a variety of oxygen‐sensitive probes, combining fluorescent NPs and phosphorescent acceptor dye, have been reported mainly based on conjugated polymer NPs.^[^ ^281^, ^282^ ^]^ The examples of oxygen probes based on dye‐loaded silica,^[^ ^283^ ^]^ polymeric,^[^ ^257^, ^284^ ^]^ and supramolecular NPs^[^ ^285^ ^]^ are more rare. Thus, in case of silica NPs, coumarin dye was used as FRET donor and reference, while oxygen‐sensitive porphyrin dye was used as an acceptor (**Figure** **15**a).^[^ ^283^ ^]^ However, loading of coumarin at relatively low loading ratio of 0.25 wt% was used for the coumarin donor, because of ACQ at higher loadings. The co‐encapsulation of this donor with 0.25 wt% of TPP led to 92% FRET efficiency and oxygen sensitive dual emission that was successfully applied for oxygen sensing in cells (Figure 15b–d). However, the antenna effect in this system is expected to be low because of low donor/acceptor ratio. In another report, Klimant et al. used energy transfer from LH dye‐doped polymer film to oxygen‐sensitive dye for oxygen sensing.^[^ ^286^ ^]^

Our group contributed to development of oxygen nanosensor that exploited light harvesting in dye‐loaded polymeric NPs. Here, more than 2000 blue cyanine dyes with bulky counterion were encapsulated with 20 phosphorescent oxygen‐sensitive platinum octaethylporphyrin (PtOEP) acceptor dyes (Figure 15e).^[^ ^257^ ^]^ The obtained NPs were ≈70‐fold brighter than QD‐525 at 395 nm excitation. The obtained system showed dual emission, where the relative intensity of the acceptor versus donors varied as a function of oxygen content (Figure 15f,g). Due to light‐harvesting from the donors, the acceptor emission was amplified 60‐fold, which corresponded to 1200 PtOEP dyes. This signal amplification allowed the use of low concentration of nanosensors and their encapsulated PtOEP units and thus allowed sensing of oxygen gradients in cell culture with minimal phototoxicity (Figure 15h).^[^ ^257^ ^]^

The detection of reactive oxygen and nitrogen species (ROS and RNS), such hydrogen peroxide (H2O2), superoxide (O_2_^‐^), hydroxyl radical (OH.), peroxynitrite (ONOO^‐^) is important for monitoring a variety of pathologies, including inflammation, allergic reactions, senescence, neurodegeneration, etc.^[^ ^287^ ^]^ Therefore, significant efforts were focused on nanomaterials for ROS and RNS sensing.^[^ ^288^ ^]^ With the LH properties of the NPs, the fluorescence signal of ROS and RNS probes can be amplified for better sensitivity.^[^ ^23^, ^289^ ^]^ Similarity to oxygen nanoprobes, these systems enable robust ratiometric measurements.

Thus, Feng et al. developed a polymeric self‐assembled FRET nanosensor to detect H_2_O_2_ using a 4‐carboxyl‐3‐fluorophenylboronic acid‐functionalized Alizarin Red S as a FRET acceptor.^[^ ^290^ ^]^ The nanoprobe was assembled from an amphiphilic polymer containing 7‐hydroxycourmain‐3‐carboxylic acid, which served as an FRET donor for the H_2_O_2_‐sensitive acceptor. The cleavage of boronic acid group by H_2_O_2_ lead to change in the fluorescence ratio between 7‐hydroxycourmain‐3‐carboxylic acid and Alizarin Red S, which was used for ratiometric detection of H_2_O_2_ in biological systems. Ding and Tang et al. reported a nanoprobe based on AIE. Here, they made a TPE based self‐assembled NPs with lipid‐PEG surfactant.^[^ ^291^ ^]^ The nanoprobe was not fluorescent in aqueous solution but got highly emissive after the reaction with peroxynitrate. This nanoprobe featured limit of detection (LOD) value of about 100 µM.

In past few decades there was extensive research on fluorescent sensors for neurotransmitters for early detection of neurodegenerative diseases.^[^ ^292^ ^]^ Fang et al has described a dopamine sensor with self‐assembled J‐aggregates of lithocholic acid (LCA) and 3,3′‐dipropylthiadicarbocyanine iodide (DiSC_3_(5)) in aqueous solution.^[^ ^293^ ^]^ This assembly forms nanotubes with 20 nm thick walls. The DiSC_3_(5)/LCA form J‐aggregate by ionic interactions confirmed by the absorption band at 711 nm in aqueous solution which was not there in monomeric form in solution. Dopamine is known to get oxidized to its quinone (electron acceptor) form, which can quench fluorescence signal by electron transfer. When this quinone derivative of dopamine adsorbed on nanotubes by π‐π interactions, it quenched ≈250 donor cyanine dyes due to delocalized excitons (by EET) within J‐aggregates. This system showed limit of detection (LOD) value as low as 0.4 nM. This low value of LOD can be explained by the LH nature of the nanoprobe, where one molecular recognition leads to quenching of a large number of dyes coupled to fast EET.^[^ ^293^ ^]^

Other important examples include sensors of ions, which are ubiquitous in biological systems.^[^ ^294^ ^]^ In an early report, Méallet‐Renault descried BODIPY‐loaded polymeric NPs, bearing an chelating ligand (cyclam) at the surface.^[^ ^295^ ^]^ Complexation of Cu^2+^ led to efficient quenching of large ensemble of BODIPY dyes, leading to effective quenching by energy transfer of ≈44 donor dyes by a single acceptor metal complex. This effective quenching enabled amplified sensing of Cu^2+^ with clear signal observed already at 5 nM cation concentration. Rampazzo et al. reported a nice application of PluS NPs loaded with coumarin dyes (donors) for amplified fluorescence sensing of Cu^+^.^[^ ^296^ ^]^ A BODIPY‐based sensor of Cu^+^ (acceptor) was synthesized and hosted in the shell of the NPs at stoichiometric ratio of NP: dye of 1:1. The BODIPY sensor dye can selectively form complex with Cu^+^, which increases the extinction coefficient of the BODIPY dye resulting an increase in FRET efficiency (80%). Here, this energy transfer occurred from 10 coumarin dyes to a single BODIPY sensor, which is attached to the shell of the NPs. In comparison with just the free sensor, the energy transfer from NPs enhances the emission signal by about 35% at the same concentration of the acceptor dye which confirms its LH antenna efficiency. More recently, sulfonatocalix[4]arene amphiphiles were used to assemble AIEgen TPE‐4Py into light‐harvesting NPs.^[^ ^297^ ^]^ In the presence of acceptor (cationic coumarin derivative) at donor: acceptor ratio 56:1, this system showed efficient energy transfer (75.2%) and antenna effect of 28.1, which enabled amplified detection of sulfite in solution and live cells. In another recent study, the light‐harvesting principle was applied for improving sensitivity of ion‐sensing poly(vinyl chloride) PVC membranes.^[^ ^298^ ^]^ Here, anionic pyrene derivative paired with bulky hydrophobic counterion lipophilic phosphonium was loaded inside PVC as energy donor, while fluorescent sensing dye, fluorescein dodecyl ester, paired with the same hydrophobic counterion was loaded as acceptor at donor acceptor ratio 200:1. The anion selectivity was provided by an chloride ionophore IV. The obtained sensor showed 22‐fold improvement in the sensitivity due to signal amplification by light harvesting.

There are other examples of LHS as small molecule sensors, however for the sake of review size they are not exhaustively covered here. Nevertheless, we have to admit that this direction is still underexplored especially for organic LHS and we expect that that further efforts will be needed to exploit in full the potential of LHS in development of ultrasensitive assays for small molecules and ions.

### 5.3 Detection of Biomolecules

**Figure 16:**
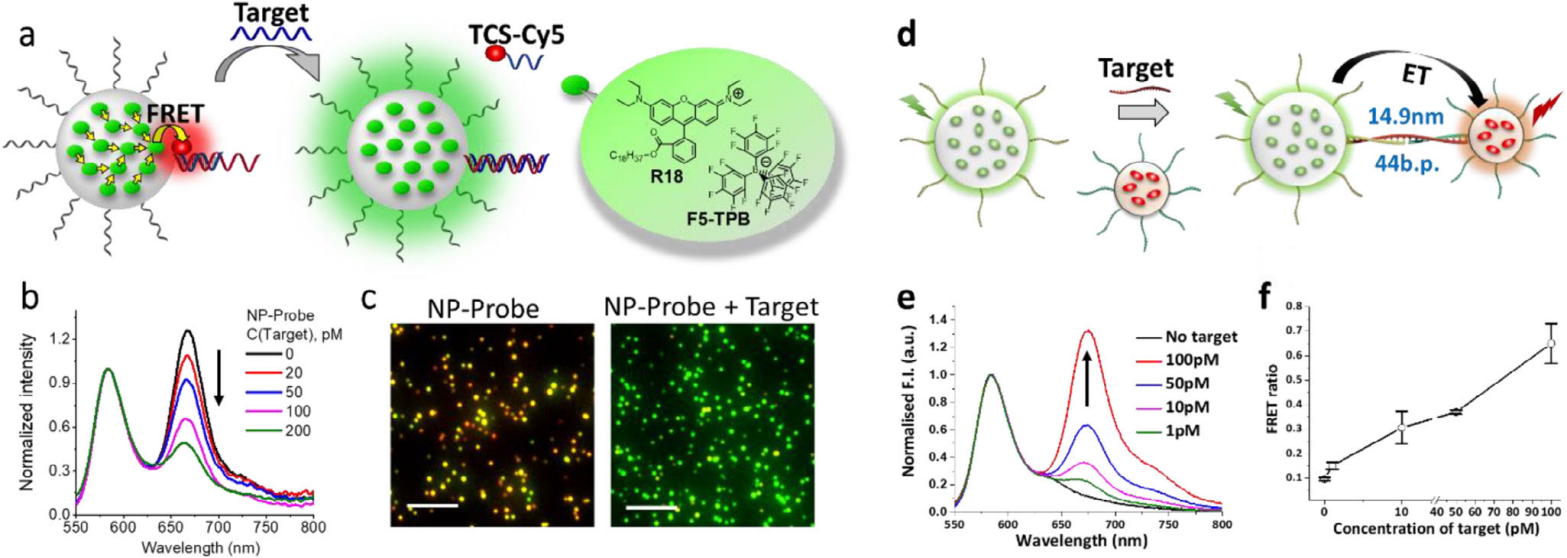
a) Concept of polymeric nanoantenna for amplified detection of nucleic acids. The dye‐loaded nanoparticle is schematically presented, bearing noncoding (in gray) and capture (dark red) oligonucleotides. Yellow arrows schematically show the light‐harvesting process toward FRET acceptor at the surface of nanoparticle. Target NA displaces short oligonucleotide with FRET acceptor (TCS‐Cy5) from the NP surface, resulting in the FRET loss, so that the emission of the probe switches from red (acceptor) to green (donor). b) Fluorescence spectra of NP‐probe (100 pM of TCS‐Cy5) after incubation for with increasing concentration of the DNA target (fragment encoding survivin). c) Response of surface‐immobilized NP‐probe to the DNA target at the single‐particle level of the NP‐probe (left), nanoprobe after incubation with 100 pM survivin DNA target for 20 h at 4 °C (right). The images represent a merge of green (donor) and red (acceptor) channels. The excitation wavelength was at 550 nm with 0.4 W cm^−2^ power density. Scale bar: 5 µm. Reproduced with permission.^[^ ^71^ ^]^ Copyright 2018, American Chemical Society. d) Response of the NP–NP FRET assay to the nucleic acid target. Schematic representation of DNA‐modified NPs loaded with donor (50 wt% of R18/F5‐TPB versus polymer) and acceptor (30 wt% of DiD/F5‐TPB versus polymer) dyes at surface‐to‐surface distance of 14.9 nm. The target DNA is represented in red color. e) Normalized steady‐state fluorescence spectra and (f) corresponding FRET ratios for the donor (8 pM) and acceptor (16 pM) NPs with varied concentration of the nucleic acid target (fragment encoding survivin). HEPES (Mis‐B) buffer containing total RNA extract from cells (0.01 g L^−1^) was used. Reproduced with permission.^[^ ^262^ ^]^ Copyright 2023, John Wiley and Sons.

Detection of nucleic acids, especially RNA, is particularly attractive for molecular diagnostics of cancer and viral diseases because a large number of RNA disease markers, such as microRNA, long non‐coding RNA, viral RNA, etc have already been identified. Detection of RNA is particularly challenging because of their very low abundance in biological samples. Therefore, the standard methods of RNA detection require molecular amplification, such as RT‐PCR and isothermal methods. The use of LHS for direct amplification of the signal offers solutions for direct detection of nucleic acids without molecular amplification. Our group has developed DNA‐functionalized dye‐loaded polymeric nanoparticles nanoantenna for amplified detection of DNAs. For this, we grafted DNA on the surface of 40 nm dye‐loaded NPs. Efficient energy transfer to just few acceptors annealed at the NP surface allowed very sensitive and selective amplified detection of DNA with LOD value of 0.25 pM on surfaces and 5 pM in solution (**Figure** **16**a–c).^[^ ^71^ ^]^ Then by increasing the hydrophobicity of the polymer, the dye loading of the NPs could be increased to 33 wt.% (versus total mass of the system) while preserving high fluorescence quantum yield. These systems, containing 600–1000 donor (R18/F5‐TPB) dyes, underwent ≈50% FRET to a single acceptor (ATTO665), which resulted in the signal amplification factor (antenna effect) of 250.^[^ ^252^ ^]^ They showed capacity to detect single DNA hybridization events, which was observed as ON/OFF switching of FRET in single NPs. The LOD of these nanoprobes in solution was 2 pM, which was limited by the kinetics of hybridization.^[^ ^252^ ^]^ The same concept was then extended to microRNA detection in cell extracts with LOD value of 1.3 pM.^[^ ^255^ ^]^ For comparison, conjugated polymer NPs were recently developed as light‐harvesting systems for amplified detection of microRNA using similar strand‐displacement principle.^[^ ^299^ ^]^ The achieved LOD was similar (1.7 pM), although the signal amplification was somewhat smaller (37‐fold). A similar principle of microRNA detection was also realized with light‐harvesting nanoantenna based on inorganic nanoparticles assembled from oligomeric silsesquioxane (POSS) bearing dansyl as a donor dye.^[^ ^300^ ^]^ It was claimed that the obtained DNA‐functionalized NPs containing ≈4000 dyes can undergo nearly quantitative FRET to 2 acceptors (Rose Bengal) hybridized at the surface, which resulted in the signal amplification of 70.5, important for amplified detection of microRNA in biological samples.

Recently, we developed an alternative approach that turns on FRET between two particles in the presence of the nucleic acid target.^[^ ^262^ ^]^ As mentioned above, FRET between the two dye‐loaded NPs can go beyond the Forster radius, which allowed us to use a DNA sandwich assay, where the target brings together two NPs at a distance of ≈15 nm (Figure 16d–f). The approach enabled detection of DNA and RNA fragments with sensitivity down to 0.36 pM in solution and with shorter incubation time, which constituted significant improvement compared to previously reported strain displacement assay.

Examples of protein detection using FRET are rarer probably because they create relatively long distances between donor and acceptor units. Nevertheless, one should mention a recent report, which used dye‐loaded polymeric NPs with thin shell, which was used as a light‐harvesting donor particle to achieve Enhanced FRET Imaging of Protein‐Specific Sialylation.^[^ ^301^ ^]^ These NPs loaded with Cy3 dye and functionalized with Sgc8 DNA aptamer could specifically bind to cell surface target protein, protein tyrosine kinase‐7. Due to thin zwitterionic shell, these NPs ensured efficient FRET to Cy5‐labelled sialic acid attached to this membrane protein, thus enhancing signal from the labelled protein.

## 6 Conclusions and Outlook

Natural light‐harvesting systems (LHS) are remarkable optical nano‐devices that collect the energy of sunlight and use it for photosynthesis of oxygen and organic matter on our plant. For decades, they inspired researchers to reproduce them in the laboratory in form of functional molecules and nanomaterials – artificial light harvesting systems. This review aims to summarize major examples of organic light‐harvesting systems based on dyes and analyze their design principles, optical properties and performance. The dye‐based systems are in focus here, because Nature selected dyes (porphyrin derivatives) to create LHS of plants and bacteria. The research on artificial LHS continue attracting strong attention because, on the one hand, it opens the route to development alternative energy sources in form of photovoltaic and photosynthetic devices. On the other hand, LHS offers a unique route to signal amplification for chemical and biological sensing, which is particularly highlighted in the present review.

In this work, we analyzed the basic concepts of multi‐chromophore systems and divide them into major classes. The theory of H‐ and J‐aggregates is fundamental here because it provides the basic understanding of how the fluorophores should be organized in order to control their absorption, emission and energy transfer properties. The major problem that has to be addressed in dye‐based LHS, is aggregation‐caused quenching (ACQ), which leads to the energy losses impacting the performance of the devices. Therefore, we analyzed the major principles of preventing ACQ, notably by using bulky substituents, bulky counterions or AIE concepts. The key process taking place in LHS is excitation energy transfer (EET), which ensures transport of the energy though multiple donor chromophores of the LH antenna toward the photo‐reaction center (acceptor). One should note that in vast majority of artificial LHS, the donor and acceptor are fluorescent dyes, which are suitable for observation and quantification of LH phenomena. Moreover, fluorescent dyes undergo minimal non‐radiativee decay, which allows the major part of energy to be transferred instead of being dissipated. The Förster and Dexter theories are the two pillars on which the current understanding of EET stands. Moreover, the theory of coherence of multiple chromophores emerged in recent literature to explain ultrafast EET in LHS. One should note that efficient LHS must be built of densely packed and properly organized chromophores that ensures fast EET on the ps‐fs time scale with minimal energy losses by ACQ, thus allowing long exciton migration distances in the 10–100 nm range. These systems with high dye cooperativity allow efficient transfer from large number of donor dyes of the LH antenna to a single energy acceptor, leading to optical amplification, so called antenna effect. While majority of examples of LH systems present antenna effect on the order of 10, some recent examples show the possibility to achieve antenna effects on the order of 100–1000.

Next, we provide detailed analysis of individual classes of artificial LHS. The key characteristics for the selected examples are summarized in **Table** **1**. We should note that conjugated polymers were excluded from the analysis because they have already been extensively reviewed and because they are usually not built by assembly of individual dyes. We start with classical examples of covalent systems, such as dendrimers and macrocycles of dyes. They symbolize the power of organic synthetic chemistry, allowing generation of molecules, containing arrays of chromophores, mainly in the range from 4 to 32. Even though these examples are crucial for fundamental research on the mechanisms of LHS, their performance in terms of AE (Table 1) is limited by the number of donor dyes combined by covalent bonds within the molecule. Therefore, we make a special focus on supramolecular systems – nanomaterials assembled mainly by non‐covalent interactions of large number of chromophores. Here, we start with examples of classical dyes, such as porphyrins (closest analogues of natural LHS), perylene bisimides, etc, with particular focus on their J‐aggregates. These systems exhibited fast EET (≤1 ps) and large exciton diffusion lengths (≥100 nm). In these systems a single acceptor could trigger an ON/OFF switching of hundreds of dyes within J‐aggregate confirming efficient EET. Further studies would be needed to evaluate their acceptor amplification (antenna effect) capabilities. AIE nanomaterials are particularly promising LHS, because AIE concept provides an elegant and universal solution to ACQ, allowing tight packing of propeller‐like chromophores. These systems are generally characterized by high QYs and energy transfer efficiencies. The antenna effect in these systems reach relatively high values on the order of 100 (Table 1). However, further enhancement of their performance would require overcoming major challenges, such as increasing extinction coefficient of the AIE chromophores for more efficient light absorption and decreasing the Stokes shift to ensure faster EET. Another remarkable class of LHS are metal‐organic frameworks (MOFs). Metal coordination in MOFs enables superior control of chromophore assembly. The latter yields MOF materials with high QY and efficient EET. Nevertheless, this research direction is still emerging and more research is needed to obtain LHS with high performance in terms of antenna effect. Next, ion‐associated materials constitute an important class of LHS that emerged in the last decade. They use a concept of bulky hydrophobic counterion that serves as a spacer between chromophores, preventing ACQ and ensuring very short dye‐dye spacing around 1 nm. The key advantage of these systems is that they employ cationic cyanines and rhodamines, which are the most performant dyes in terms of extinction coefficient and fluorescence QY, while exhibiting small Stokes shift. These ion‐associated materials present high QY values together with efficient EET, yielding materials with antenna effect reaching rather high values around 200 (Table 1).

**Table 1:** Optical properties, composition and antenna effect of selected example of dye‐based LH nanomaterials.

Type of LH antenna	Dyes used: donor/acceptor	λ_ex_ [nm]	λ_ex_ [nm]	Donor/Acceptor ratio	FRET efficiency [%]	Antenna effect	Refs.
Covalent systems	Coumarin / coumarin‐343	N/A	N/A	16/1	93	4.3	[108]
Covalent systems	Peryleneimides / terrylenediimide	495	700	8/1	quantitative	3.0	[110]
AIE	salicylaldehyde azine / Nile red	365	619	150/1	55	25.4	[164]
AIE	salicylaldehyde azine / Eosin Y	365	549	200/1	42	28	[164]
AIE	TPE / sulforhodamine 101	350	610	150/1	64	13.0	[157]
AIE	TPE‐CHO / TPE‐TCF	371	570	1000/1	32	35	[302]
AIE	TPE / NDI	390	640	500/1	58	16	[159]
AIE	TPE / anthracene derivatives	368	560	1000/1	N/A	90.4	[162]
AIE	TPE / NDI	330	640	1250/1	‐	63	[160]
AIE	p,p′,p″‐HPTPM / IR780	438	831	500/1	29	14.3	[303]
AIE	β‐CD‐modified NDI / TPE	340	480	40/1	57	13	[304]
AIE	oligo(phenylenevinylene) / Nile Red	365	540	125/1	72	32.5	[161]
AIE	BPT / DBT	365	550	350/1	60	47.8	[166]
AIE	TPE / Nile Red	365	610	200/1	74	11.5	[165]
AIE	Dimeric TPE / Nile Red	365	583	250/1	56	66	[168]
AIE	TPE derivative with metal / diketopyrrolopyrrole	361	568	12.5/1	53	12.8	[305]
Ion‐associated NPs	R18/F5‐TPB / Cy5/Cl	520	680	1000/1	50	200	[199]
Ion‐associated NPs	dicationic cyanostilbenes / Nile Red	365	655	500/1	80	150	[203]
DNA origami	Fluorescein / Cy5	450	665	8 /1	>70	8.7	[213]
DNA origami	Pyrene / Alexa 647	370	660	6/1	90	0.93	[209]
DNA origami	Cy3 / Cy5	521	660	6 /1	≈30	<2	[212]
DNA origami	YOYO1 / Cy3	440	570	10/1	52	6.0	[221]
DNA origami	YO‐PRO1 / porphyrin	491	700	20/1	49	12	[97]
DNA/Supramolecular polymers	phenanthrene oligomers / Cy3	322	570	33/1	43	23	[222]
Protein self‐assembly	Oregon green‐488 / Alexa‐594	495	597	101/1	34	11.2	[223]
Peptide self‐assembly	Pyrene / naphthalene monoimide	335	520	25/1	72	15.8	[226]
Micelles	DPA / Eosin Y	450	550	48/1	50	7.6	[228]
Micelles	Cy3 / Cy5	550	670	50/1	69	21	[229]
Micelles	photophyrin and phycocyanin	420	640	179/1	80	37	[230]
Supramolecular nanofibers	thioflavin T / thiazole orange and pyronin Y	440	573	25 / 1	N/A	2.7	[306]
Supramolecular Columns	Tricyanotristyrylbenzene / Nile Red	365	640	20 000/1	40	100	[236]
Dye‐doped silica NPs	Rhodamine / Cy5	550	670	10/1	90	4.5	[243]
Dye‐loaded polymer NPs	Quaterthiophene / Nile Red	370	600	125/1	56	3.2	[307]
	R18/F5‐TPB / DiD/F5‐TPB	530	680	10 000/1	67	1150	[98]
	R18/F5‐TPB / DiD/F5‐TPB	530	680	40 000/1	23	4800	[258]
	R18/F5‐TPB / Cy5	530	680	138/1	60	58	[71]
	R18/F5‐TPB / ATTO647N	530	670	1000/1	51	209	[252]

To achieve both high control of spatial organization of dyes and nanoscale architecture of the nanomaterials, the efforts were focused on hybrid materials combining dyes with biomolecules, surfactants, polymers or inorganic matrices. Here, we first highlighted the classical early examples of LHS based on nucleic acids. DNA origami allows creation of well‐defined nanostructures, where the dyes can be precisely positioned by either covalent bonds or intercalation. A number of elegant examples of DNA origami‐based LHS were reported showing efficient energy transfer through long distances as well as impressive cascade FRET systems involving, for instance, 5 dyes of different color. They also enabled demonstration of long‐range FRET due to transfer of energy between chromophores organized in 2D planes. Nevertheless, these systems still exhibit limited values of antenna (1‐12, Table 1), probably because of relatively long dye‐dye spacing and typically 1D transfer within a DNA “wire” compared to 3D transfer in some other LHS. Proteins are also used for LHS preparation, but, paradoxically, the examples of those systems are rarer, despite the fact that Nature uses proteins for this purpose. LHS can be also built based on assembly of dyes into micelles or supramolecular polymers. These systems can feature higher antenna effect values (100, Table 1), which are determined by the aggregation number. Dye‐doped silica NPs has been also a popular nanomaterial for building LHS due to their high stability and monodispersity. However, so far, relatively low dye loading was achieved in these systems, which limit the currently achieved values of antenna effect (Table 1). Therefore, the search for new dye loading concepts with minimized ACQ will be important for silica NPs. On the other hand, dye‐loaded polymeric NPs seem to meet best all the requirements to obtain high‐performance LHS. Indeed, using the concept of bulky hydrophobic counterion, exceptionally high dye loading can be achieved (23–50 wt% versus total NP mass), while preserving good QY values (30–40%). In this case, the dye‐dye distance becomes really small (1–2 nm), allowing ultrafast EET down to 30 fs with exciton diffusion distances reaching ≈100 nm. Therefore, dye‐loaded polymeric NPs exhibit the highest antenna effect values obtained so far: 1150, reported in 2017, and 4800, reported in 2024 (Table 1). These values are even higher than those achieved for the best plasmonic systems, applied to good fluorophores (300) located between two gold NPs with help of DNA origami. Moreover, combination of two dye‐loaded polymeric NPs allows breaking Forster distance barrier, which reveals power four dependence on the donor‐acceptor distance, similar to that reported for transfer between 2D planes in DNA origami. Nevertheless, the current limitation of dye‐loaded polymeric LHS is their whole‐particle blinking caused by trapping of the rapidly migrating excitation energy by the transient dark states. Another limitation is the poor understanding of dye/counterion organization (packing) in pure salts or inside polymeric NPs, which should be in the future addressed by appropriate theoretical and experimental methods.

The signal amplification provided by LHS is a fruitful mechanism for development of fluorescent nanoprobes for amplified sensing. In contrast to methods of molecular amplification (such as PCR), LHS enables direct physical amplification, allowing much simpler detection of analytes. Examples selected in this review include detection of small molecules, such as oxygen, reactive oxygen and nitrogen species and neurotransmitters. LHS allows increasing sensitivity of these nanosensors and decreasing the toxicity effects, in particular, for oxygen sensing that is associated with singlet oxygen generation. However, this signal amplification strategy remains underexplored in the design of nanoprobes for small molecules, and we foresee many more examples of amplified nanosensors to appear in the near future. Moreover, LHS are particularly attractive for amplified detection of large biomolecules, such as nucleic acids, which are generally present at low concentrations in biological fluids. Nanoprobes based on LHS enable direct quantitative detection of RNA and DNA markers of diseases, without PCR‐based amplification, which opens the route to rapid point‐of‐care molecular diagnostics of cancer and viral diseases. Despite the fact that LHS‐based nanoprobes enable detection of single‐molecule hybridization events, their sensitivity in solution remains limited to ≈1 pM concentration. The reason stems from slow diffusion limited kinetics of analyte‐probe interaction when the two interacting partners are too diluted. Moreover, LHS systems are less adapted for protein sensing because the diameter of proteins, especially antibodies, can be larger than the Förster radius. The search for new sensing schemes should continue in order to take full advantage from amplification capacity of LHS for high‐sensitivity detection of large biomolecules.

Overall, we consider that the research on LHS enters into a new exciting phase, due to emerging possibilities to boost their performance through appropriate design of dye‐based nanomaterials. On the other hand, LHS opens new horizons in amplified sensing that in the future could result in next generation devices for point‐ofcare diagnostics and molecular imaging in biological and biomedical context. Moreover, the applications of LH materials go well beyond biosensing, which includes exciting opportunities in solar energy conversion for photovoltaics, photocatalysis and photosynthesis, such as water splitting and CO_2_ reduction as well as optoelectronic and neuromorphic devices for light detection and artificial vision, smart textiles and wearables, etc. Here, with proper design, organic LH materials will offer unique opportunities in terms of LH performance, mechanical properties and environmental safety, complementary to what can be offered by inorganic LH materials.

## Conflict of Interest

A.S.K. is a co‐inventor of filed patent applications on fluorescent polymeric nanoparticles and cofounder of BrightSens Diagnostics SAS. Other co‐authors have no conflict of interest to disclose.
